# Cetacean distribution models based on visual and passive acoustic data

**DOI:** 10.1038/s41598-021-87577-1

**Published:** 2021-04-15

**Authors:** Kaitlin E. Frasier, Lance P. Garrison, Melissa S. Soldevilla, Sean M. Wiggins, John A. Hildebrand

**Affiliations:** 1 Scripps Institution of Oceanography, La Jolla, CA USA; 2 Protected Resources and Biodiversity Division, NOAA NMFS Southeast Fisheries Science Center, Miami, FL USA

**Keywords:** Biooceanography, Ecological modelling, Ecology, Biogeography, Acoustics, Biological models, Marine biology

## Abstract

Distribution models are needed to understand spatiotemporal patterns in cetacean occurrence and to mitigate anthropogenic impacts. Shipboard line-transect visual surveys are the standard method for estimating abundance and describing the distributions of cetacean populations. Ship-board surveys provide high spatial resolution but lack temporal resolution and seasonal coverage. Stationary passive acoustic monitoring (PAM) employs acoustic sensors to sample point locations nearly continuously, providing high temporal resolution in local habitats across days, seasons and years. To evaluate whether cross-platform data synthesis can improve distribution predictions, models were developed for Cuvier’s beaked whales, sperm whales, and Risso’s dolphins in the oceanic Gulf of Mexico using two different methods: generalized additive models and neural networks. Neural networks were able to learn unspecified interactions between drivers. Models that incorporated PAM datasets out-performed models trained on visual data alone, and joint models performed best in two out of three cases. The modeling results suggest that, when taken together, multiple species distribution models using a variety of data types may support conservation and management of Gulf of Mexico cetacean populations by improving the understanding of temporal and spatial species distribution trends.

## Introduction

The oceanic Gulf of Mexico (GOM) provides habitat for a diverse array of pelagic cetaceans including sperm whales, beaked whales, and a variety of delphinids^1^. These oceanic species, found beyond the continental shelf, are thought to represent the majority of GOM cetaceans in terms of total numbers and species richness^2,3^, however the temporal trends and spatial distributions of these populations are poorly understood due to the many challenges of offshore cetacean monitoring. The dynamic oceanography of the GOM is uniquely driven by the Loop Current, which sheds large anticyclonic eddies that drive vertical mixing as they drift across the basin^4^. The northern GOM is also influenced by freshwater and terrestrial inputs from the Mississippi River as well as from surrounding bays and estuaries. Variability due to these environmental factors may influence and drive changes in cetacean distributions^5,6^.

Shipboard and aerial line-transect visual surveys are the standard method for estimating densities and describing the distributions of cetacean populations^5,7–11^, relying on animal sightings when they surface to breathe. These surveys provide broad spatial coverage of a region at a conceptual snapshot in time, and observers can provide visual species identifications and group size estimates. Some temporal coverage is also obtained if multiple surveys are combined over many seasons and years. However, visual methods are resource-intensive, requiring expensive vessel/aircraft and personnel time. They also rely on fair weather conditions, restricting most survey effort to summer months, with lesser survey effort occurring in winter months^3,12,13^. Furthermore, visual surveys can be challenging for visually cryptic, deep diving species, such as beaked whales and *Kogia* spp., which spend little time at the sea surface.

Static passive acoustic monitoring (PAM) provides a complementary modality for cetacean monitoring; this approach employs acoustic sensors at fixed sites and provides a nearly continuous record of animal presence^14^. Instead of visual identification, this method relies on detection of species-specific acoustic signals. We have been collecting PAM recordings in the GOM nearly continuously using fixed seafloor sensors since 2010^15,16^. The time series from acoustic monitoring sites provide excellent temporal coverage, operating continuously regardless of weather conditions or time of day. However, spatial coverage is limited, because sensor locations are fixed and detection ranges are restricted by the acoustic characteristics of the environment and vocalizations monitored^17,18^. Acoustic species identification is well established for many toothed whale species; however, the ability to acoustically distinguish some delphinid species remains limited. And while methods for group size estimation for odontocetes from acoustics exist, current methods rely on labor-intensive tracking of individual click trains^19,20^.

Visual survey and PAM datasets have been used independently to predict cetacean distributions or occurrence across space and time under varying oceanographic conditions^2,3,21^; however, the limitations of each survey modality respectively result in an incomplete picture of spatiotemporal occurrence of cetaceans. We describe a pilot study examining the predictive ability of visual and acoustic data independently, and the feasibility of combining visual and acoustic data along with environmental measurements into joint distribution models capable of leveraging both the spatial coverage of visual survey data and the temporal coverage of static PAM data collected in the GOM. The differences between the data types are considered, and strategies are presented for overcoming these differences, in addition to simultaneously utilizing both data types. Two modeling frameworks, generalized additive models and neural networks are evaluated as alternative implementations. Distribution models from both frameworks using individual and combined sampling methodologies were developed for three species including Cuvier’s beaked whale (*Ziphius cavirostris*), sperm whales (*Physeter macrocephalus*), and Risso’s dolphins (*Grampus griseus*). Potential for further development, data collection needs, and model improvements based on this pilot study are discussed.

## Methods

Distribution models were produced for Cuvier’s beaked whale, sperm whale, and Risso’s dolphin based on the availability of both visually and acoustically distinctive features for species detection such as size, body markings and shape, and on temporal-spectral characteristics of their echolocation clicks^22–24^.

### Visual surveys

**Figure 1:**
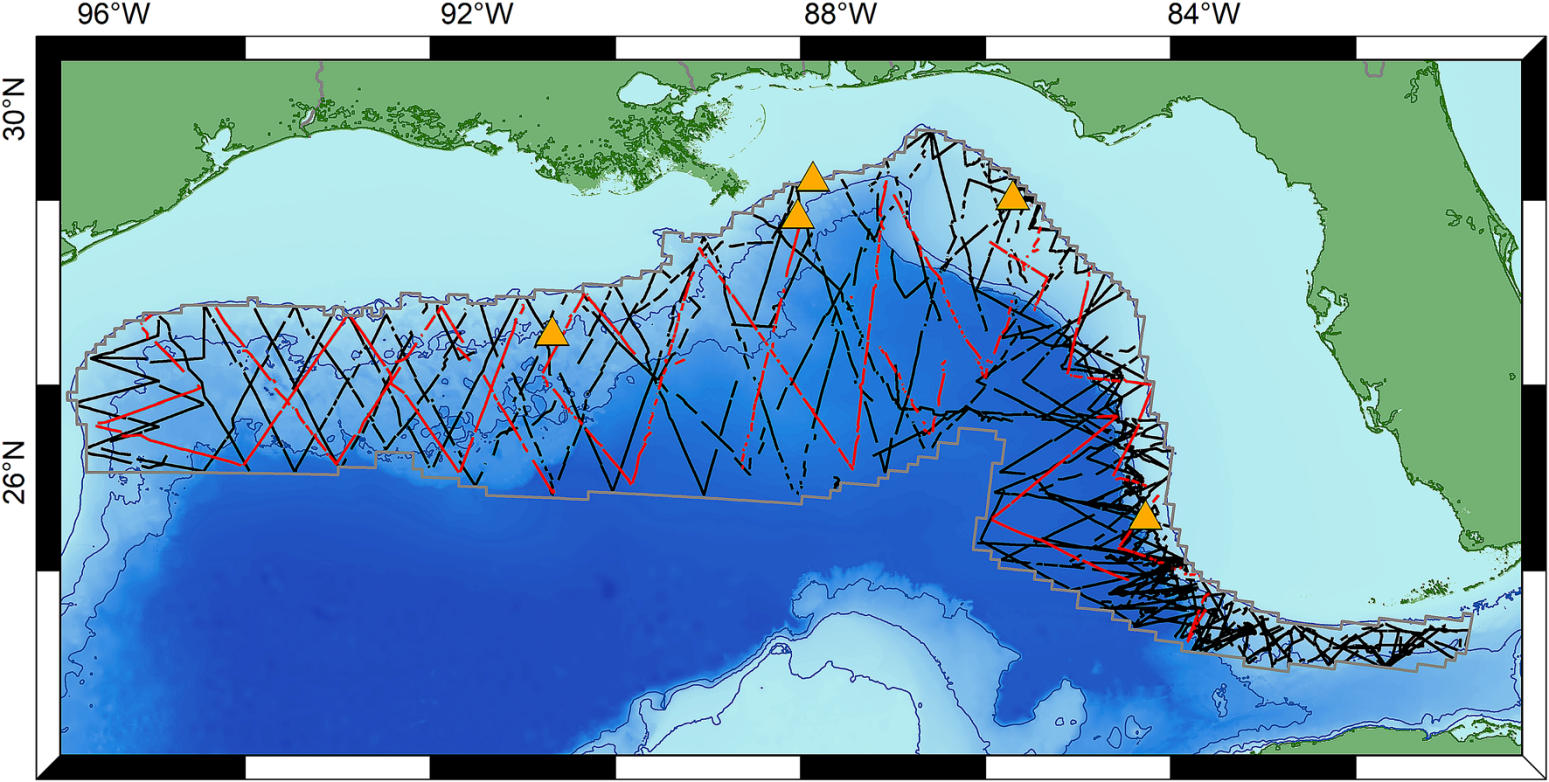
Map of GOM visual survey effort for five NOAA cruises between 2003 and 2014 (lines) and passive acoustic monitoring locations (orange triangles). The 2009 cruise effort (red lines) was used for model testing. Track lines for all other years, used for model training, are shown in black. The gray outline shows the extent of the modeled region, a pelagic area encompassing depths greater than 200 m within the US EEZ. Bathymetric contours (blue lines) are shown for the 200 m, 1000 m and 2000 m contours (Map created using ArcGIS software by ESRI^29^).

Visual survey data were collected during five cruises conducted by the National Oceanographic and Atmospheric Administration Southeast Fisheries Science Center (NOAA SEFSC) aboard the R/V Gordon Gunter in 2003, 2004, 2009, 2012, and 2014 (Fig. 1, Supplementary Table 1)^25^. These cruises were designed to survey the oceanic GOM; therefore, the survey area was delimited by the 200 m bathymetric contour to the north, west, and east, and by the limit of the US exclusive economic zone (EEZ) to the south. Cruises conducted in 2012 and 2014 were limited to the eastern GOM. For this study, cruise data from 2009 was used only for model testing, while other years were used for training. The 2009 data were selected for testing because the entire area was surveyed in that year, allowing model predictions to be evaluated across the full region of interest. Pre-2003 visual survey data were not used due to limited availability of environmental covariate measurements for earlier years.

Visual survey effort was conducted along transect lines with the vessel traveling at or above 18.5 km/h (10 kn). To mitigate spatial autocorrelation between successive sightings, transect lines were divided into equal length segments of 10 km or less with transect segments each representing approximately 0.5 h of survey effort. The visual survey dataset consisted of 1,956 training segments and 449 test segments. Observations were used as point estimates. Implications of this approach are considered in the discussion.

On all visual surveys, observation data were collected by one team of trained visual observers on the vessel’s flying bridge using 150 × 25 Bigeye binoculars to search for, identify, and estimate group sizes of cetaceans. All surveys operated in closing mode with the vessel departing from the track line for closer approaches to identify to the lowest possible taxonomic level and to obtain group size estimates.

Raw count data were converted into densities for each 10 km transect segment for each of the three species of interest. All sightings for each of the species of interest are shown in Supplementary Figs. 1, 3, and 5. To obtain densities using distance sampling methodologies, the best model to fit the distribution of sighting distances was selected from a range of options (half normal, hazard-rate, hazard-rate with a second order polynomial adjustment, or uniform) using AIC implemented in the R software package *mrds*^26^. The species-specific sighting probability (*P*_*vis*_) along a transect segment was given by the fitted detection function. Species-specific estimated truncation distances (or effective strip half width; *w*) were computed as the distance from the transect line within which 95% of the sightings of each species occurred (Supplementary Figs. 2, 4, and 6).

For each transect segment and species, the total area monitored visually (${A}_{Vis}$) was computed as

1 $${A}_{Vis} = 2wL$$

where *L* is the transect segment length. Animal density was calculated for all transect segments as the number of animals detected per 1000 km^2^.

Density (${\widehat{D}}_{t}^{V}$) along each visual survey transect segment *t* was calculated as.

2 $$\hat{D}_{t}^{V} = \hat{G}_{tot} /({\text{A}}_{vis} \cdot \, g\left( 0 \right) \, \cdot\hat{P}_{vis} )$$

where $\widehat{G}$_*tot*_ is the sum of the best estimate group sizes from all sightings of the species of interest along the transect segment, and *g(0)* is the probability of observing the species directly on the transect line^27^ (Supplementary Table 2). Estimation of *g(0)* typically requires survey effort using independent (double-blind) observer teams and estimates were not available for the GOM surveys; therefore *g(0)* was estimated for each species from western Atlantic surveys aboard a similarly-sized vessel (R/V *Endeavor*), as the average of *g(0)* estimates from upper and lower observation teams^28^. The upper and lower observation platforms of the *R/V Endeavor* were 17.6 and 10.2 m high respectively and a cruise speed of 10 knots. The R/V Gordon Gunter has a primary observation deck height of 13.9 m, and a survey speed of 10 knots.

### Passive acoustic monitoring

PAM data were collected from five sites in the GOM (Fig. 1) between 2011 and 2013 (Supplementary Table 2) using High-frequency Acoustic Recording Packages (HARPs)^30^. Recordings from three deep (> 1000 m bottom depth) monitoring sites were used for this study’s deepest-diving species, sperm whales and Cuvier’s beaked whales. Recordings from two additional continental shelf monitoring sites (< 300 m bottom depth) were used for the shallower diving Risso’s dolphins. PAM data collected in 2011 and 2012 were used for model training, while 2013 data were held back for model testing. The acoustic datasets for sperm whales and beaked whales consisted of 1789 training and 740 test observations. The acoustic dataset for Risso’s dolphin included data from two additional sites, and consisted of 2935 training observations and 1308 test observations.

Echolocation clicks for each target species were detected and classified in the PAM recordings^19,31,32^. Detections and classifications were manually reviewed by expert analysts to ensure low false positive and misclassification rates using *DetEdit* a custom software and graphical user interface package written in MATLAB^33^.

To reduce impacts of temporal autocorrelation between successive encounters and to avoid potential effects of diel variability in echolocation rates (e.g., Risso’s dolphin detections primarily occur during nocturnal foraging), the acoustic detections were divided into daily bins. For all days with 24 h of recording effort, estimated density was computed for each species of interest at each site. Estimated densities for each species (${\widehat{D}}_{kt}^{A}$) at acoustic monitoring site *k* on day *t* were computed using a group-counting approach

3 $${\widehat{D}}_{kt}^{A}=\frac{{n}_{kt} (1-{\widehat{c}}_{k}) \widehat{s}}{{\uppi w}^{2} {\widehat{P}}_{k} {\widehat{P}}_{v}{ T}_{kt}}$$

where ${n}_{kt}$ is the number of time intervals (5 min windows) during which groups were detected at site *k* on day *t*, $\widehat{s}$ is the average estimated group size (obtained from the visual survey estimates), and ${\widehat{c}}_{k}$ is the site-specific false positive rate for group detections. ${\widehat{P}}_{k}$ is the estimated probability of detecting a group within the maximum horizontal detection range *w*, ${\widehat{P}}_{v}$ is the probability of a group vocally active in a 5-min window, and ${T}_{kt}$ represents the total number of time intervals sampled within each day^15,34^ (Supplementary Table 4). Five minute windows were selected as an intermediate time period, long enough that the probability of a group vocalizing at some point within the window is high, but short enough that the probability of a group leaving or entering the acoustic detection area is low. Methods for density estimation are detailed in^17,19,31^ for Cuvier’s beaked whales, sperm whales and Risso’s dolphins respectively, and key parameters are listed in Supplementary Table 4.

### Environmental parameters

Environmental data were accessed through the Marine Geospatial Ecology Toolkit^35^ in ArcGIS, and through the Physical Oceanography Distributed Active Archive Center (PODAAC) maintained by the Jet Propulsion Laboratory (JPL, California Institute of Technology; https://podaac.jpl.nasa.gov/dataaccess last accessed: May 15, 2017). These data products were selected based on their spatial and temporal coverage and resolution. The same products were used for all years and survey methods. Nine environmental explanatory covariates were included in models: Sea surface height (SSH), sea surface temperature (SST), surface chlorophyll A concentration (CHL), mixed layer depth (MLD), upwelling speed at 50 m (Upwell), surface salinity (SAL), surface current magnitude (CUR), distance to nearest cyclonic eddy (+ Eddy) and distance to nearest anti-cyclonic eddy (− Eddy) (Table 1). Distances to Eddies were computed from daily rasters of sea surface height. Spatial resolution varied from 1/25 to 1/5 degrees and temporal resolution ranged from daily estimates to eight day averages. Data gaps were minimized in these products by either multi-day averaging or spatial interpolation. The nearest environmental parameter estimates in space and time were selected for each density estimate.

**Table 1:** Comparison of environmental variability observed in the visual survey and PAM datasets.

Oceanographic variable	Survey method	Mean [5th, 95th percentile]	Data source	Spatial resolution	Temporal resolution
Sea surface height anomaly (m) (Abbr.: SSH)	Visual acoustic	0.06 [− 0.20, 0.44] 0.06 [− 0.08, 0.20]	JPL^36^	1/6 degree	5 day time averaged product
Sea surface temperature (°C) (Abbr.: SST)	Visual acoustic	28.39 [23.22, 30.41] 25.57 [20.46, 30.17]	GHRSST Level 4^37^	1/5 degree*	Daily
Chlorophyll A (Abbr: CHL)	Visual acoustic	0.196 [0.062, 0.525] 0.678 [0.102, 3.223]	NASA OceanColor Group^38^	9 km	8 day average
Mixed layer depth (m) (Abbr.: MLD)	Visual acoustic	10.9 [2.2, 29.2] 21.2 [2.4, 59.3]	HYCOM^39^	1/25 degree*	Daily^†^
Vertical water velocity at 50 m depth (m/s) (Abbr.: Upwell)	Visual acoustic	− 8.84e−06 [− 3.45e−4, 3.17e−4] − 1.89e−05 [− 3.43e−4, 2.67e−4]	HYCOM^39^	1/25 degree*	Daily^†^
Salinity (PSU) (Abbr.: SAL)	Visual acoustic	35.3 [33.8, 36.1] 34.9 [33.1, 36.2]	HYCOM^39^	1/25 degree*	Daily^†^
Surface current magnitude (Abbr.: CUR)	Visual acoustic	0.54 [0.10, 1.41] 0.29 [0.07, 0.63]	HYCOM^39^	1/25 degree*	Daily^†^
Distance to nearest cyclonic Eddy (km) (Abbr.: + Eddy)	Visual acoustic	70.9 [0.0, 185.2] 136.9 [37.1, 284.8]	Derived from JPL^36^	1/6 degree	5 day time averaged product
Distance to nearest anti-cyclonic Eddy (km) (Abbr.: − Eddy)	Visual acoustic	70.9 [0.0, 185.2] 136.9 [37.1, 284.8]	Derived from JPL^36^	1/6 degree	5 day time averaged product

Asterisks (*) indicate spatially interpolated products, and obelisks (†) indicate products which are seven day hindcasts.

Environmental covariates were limited to factors that showed variability with respect to both the visual survey and PAM datasets. Certain commonly-used environmental covariates were not used, including bottom depth, current direction, and current speed, because these parameters were strongly tied to physical location and distributions were not well-sampled by the fixed PAM sensors. Also, temporal variables such as Julian day and season were excluded because they were not well sampled by the visual survey methods. Anthropogenic drivers, such as occurrence of seismic surveys, sonar, and nearby shipping activity, can be derived from fixed PAM recordings, but these variables are not consistently available for the visual survey data so they were also excluded from all models. Some variables were highly correlated, such as surface temperature with temperatures at different depths, as well as surface salinity with salinities at different depths; therefore, only surface temperature and salinity were selected (Supplementary Fig. 7). Surface chlorophyll A concentration, mixed layer depth and current magnitudes were log-transformed to minimize skew.

**Figure 2:**
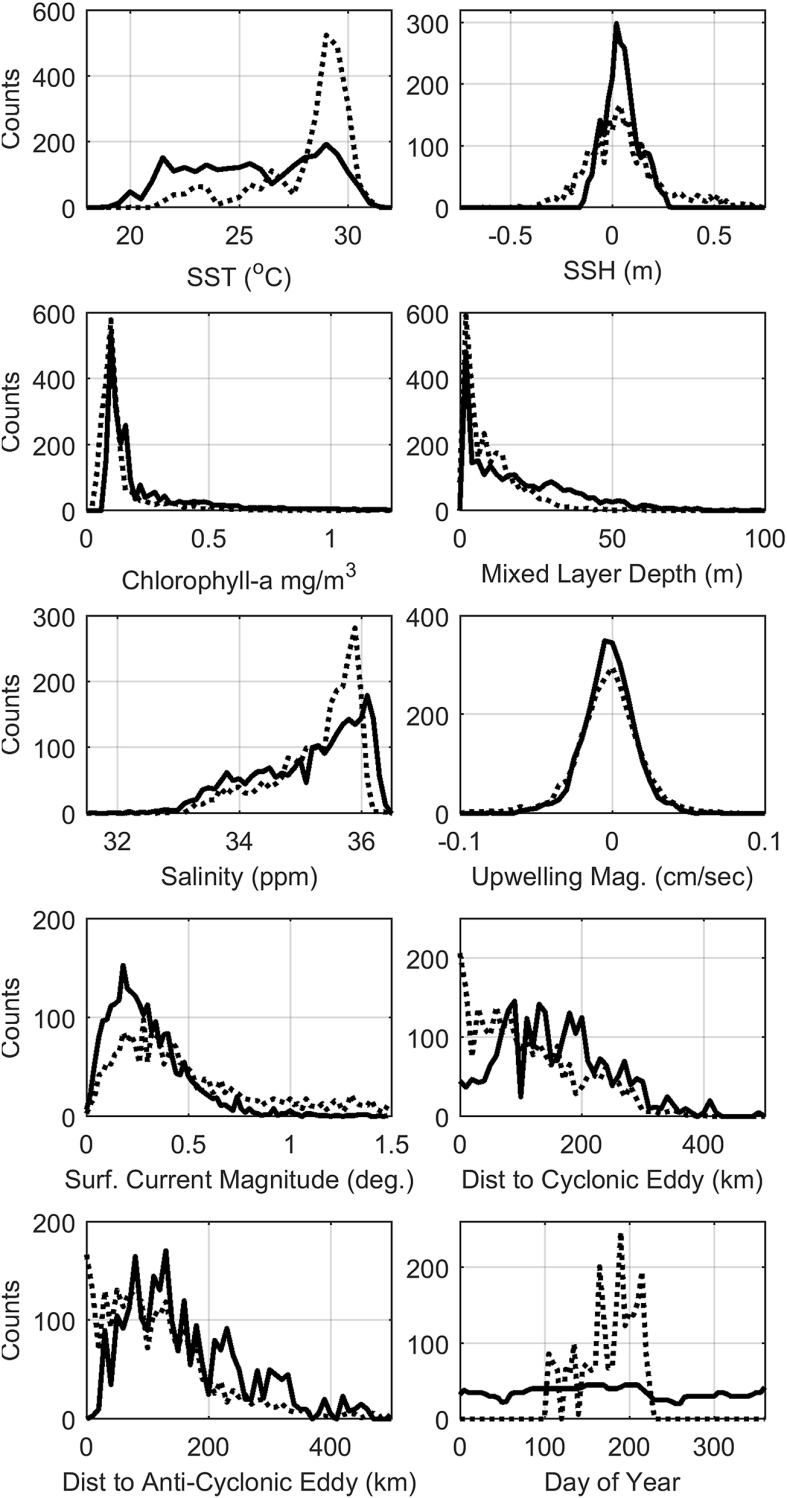
Distributions of predictor variables within the visual (dashed line) and acoustic (solid line) training datasets. Summer visual survey transects covered a wider range of mesoscale oceanographic features including currents and eddies, while year-round recording with PAM sampled a broader range of temperatures, salinities and mixed layer depths.

Visual survey track lines across the GOM surveyed within Loop Current-associated features and eddies more often than PAM sensors, and therefore traversed a wider range of sea surface heights and current magnitudes (Table 1, Fig. 2). However, by monitoring year-round, PAM sensors observed a wider range of sea surface temperatures, mixed layer depths, and chlorophyll A concentrations. Similar ranges of salinity and vertical transport velocity were observed by the two methods.

### Distribution modeling

Model inputs, code and output are available at data.gulfresearchinitiative.org/data/R6. × 805.000:0015.

#### Model frameworks

Two modeling frameworks were examined for this application: generalized additive models (GAMs) and neural networks (NNs). Density models were trained for each species within each model framework. The response variable represented the estimated density of animals present within each sample (1 day or 10 km transect segment) based on in situ observations (Eqs. 1–3), and models predicted estimated density of animals. For each of the two model types (GAM or neural network), two different models were trained using different training sets: (1) visual data, (2) acoustic data for a total of four model types trained for each species.

#### Model implementations

GAMs were constructed using the R software package *mgcv*^40,41^. GAMs were fit using a Tweedie distribution^42^ which can fit zero-inflated data better than a Poisson distribution. All predictor variables were input as smooth additive terms using the “ts” isotropic shrinkage smoother option with a three knot basis to reduce overfitting and prediction of extreme values near the edges of the training distributions. The use of ts splines allows uninformative predictor variables to be removed from the model during fitting. Following model fitting, final models were re-calculated with uninformative terms removed.

Neural networks were constructed using *avNNet* in the *caret* software package^43^, which allows repeated independent network training iterations to be run within a multi-fold framework. An average network is then computed across many trials, and model uncertainty can be estimated by calculating variability across the trials. Neural networks typically operate on scaled inputs, requiring predictor variables to be rescaled between − 1 and 1 using the maximum and minimum values observed for each predictor in the training set. Log transformation of densities improved handling of highly-skewed observations.

Each network consisted of an input layer of 9 nodes (where each node represented one predictor variable), one fully-connected hidden layer, and one output node, representing the response variable (probability of occurrence or density). Selection of the number of nodes in the hidden layer is important as it controls ability of the network to learn and store information; too few nodes would restrict the predictive power of the model, while too many would result in model over-training. The *avNNet* software package contains a simple implementation of neural networks, allowing only one hidden layer and few options for minimizing overfitting (i.e., no dropout or regularization methods). Overtraining was addressed by training networks on a range of hidden layer sizes ranging from 4 to 14 nodes and selecting the layer size that minimized prediction errors on the test data. A maximum of 500 training iterations were allowed for each network to further minimize the risk of overtraining.

For network training, node weights were randomly initialized with values from -0.7 to 0.7. A node weight decay of 1 × 10^–4^ was used to train the networks, with maximum conditional likelihood as the cost function. The *avNNet* function was used to train 25 independent networks, and calculate the average prediction on the test set for each hidden layer size. Root mean squared error (RMSE) was used to compare predictions from each hidden layer size with observations in the test data as

4 $$\mathrm{RMSE}= \sqrt{\frac{1}{N}\sum_{n=1}^{N}{({y}_{n}-{\widehat{y}}_{n})}^{2}}$$

where *N* is the number of observations in the test dataset, ${y}_{n}$ is the *n*th observation in the test dataset, and ${\widehat{y}}_{n}$ is the model estimate of the observation. The hidden layer size that minimized RMSE on the test data was selected as the best network configuration. Variability was estimated by generating predictions from each of the 25 models trained with *avNNet*, and computing the standard deviation across those predictions. Neural network plots were created using *NeuralNetTools* for R^44^.

#### Model evaluation

All models were evaluated on both visual and acoustic test sets to compare spatial and temporal predictions. The best performing model was selected by minimizing RMSE (Eq. 4)*.* RMSE favors mid-range model predictions because the squared term strongly penalizes cases in which differences between observations and model predictions are large.

## Results

### Model comparisons

Distribution models were trained and tested on the acoustic dataset (acoustic-only models), the visual dataset (visual-only models), and on the combined acoustic and visual datasets (joint models). Encounter rates and mean density estimates differed between methods (Table 2). Model fits were evaluated based on RMSE (Table 3). Species-specific findings are described in detail below.

**Table 2:** Encounter rates and mean densities of each species within the visual survey and passive acoustic monitoring training datasets. Density estimates were calculated following Eqs. (2) and (3) for visual and passive acoustic methods respectively.

Species	Encounter rate (% of observations with encounters)		Mean density (animals per 1000 km^2^)	
	Visual (% of transect segments)	Acoustic (% of days)	Visual mean (CV)	Acoustic mean (CV)
Cuvier’s beaked whale	2.0	37	0.5 (7.9)	2.8 (1.9)
Sperm whale	5.5	77	1.5 (5.2)	5.0 (1.1)
Risso’s dolphin	2.5	19	3.4 (9.7)	2.0 (4.1)

**Table 3:** Root mean squared error (RMSE) scores for density models.

Species	Acoustic only model		Visual only model		Joint model	
	GAM	NN	GAM	NN	GAM	NN
Cuvier’s beaked whale	4.97	**3.64**	5.82	4.49	**3.77**	3.71
Sperm whale	6.21	5.50	5.93	5.48	**5.61**	**5.41**
Risso’s dolphin	18.61	**17.72**	17.78	17.91	**17.63**	17.82

Best-fitting models highlighted in bold.

#### Cuvier’s beaked whale

**Figure 3:**
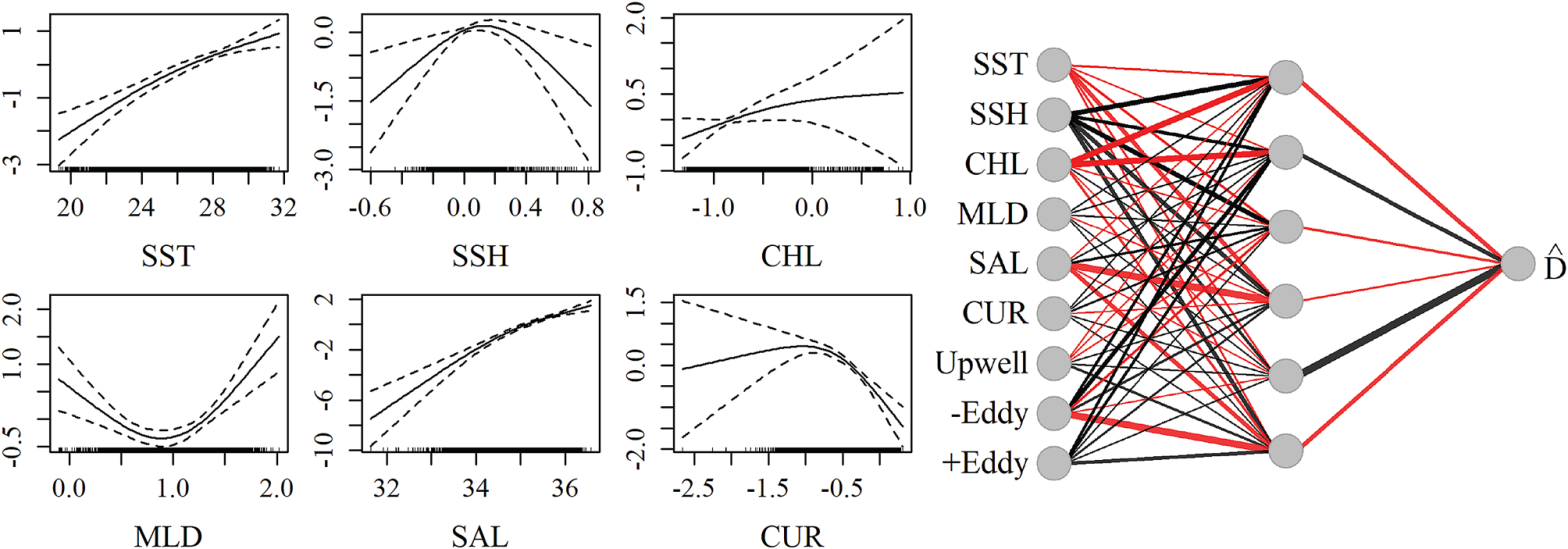
Influence of predictor variables in the best fitting Cuvier’s beaked whale models. *Left*: Smooths of significant terms in the joint GAM. *Right*: Neural network for Cuvier’s beaked whale acoustic-only model with four nodes (gray circles) in the hidden layer. Lines indicate connections between nodes, with thicker lines indicating higher weights. Black lines represent positive weights and red lines represent negative weights.

**Figure 4:**
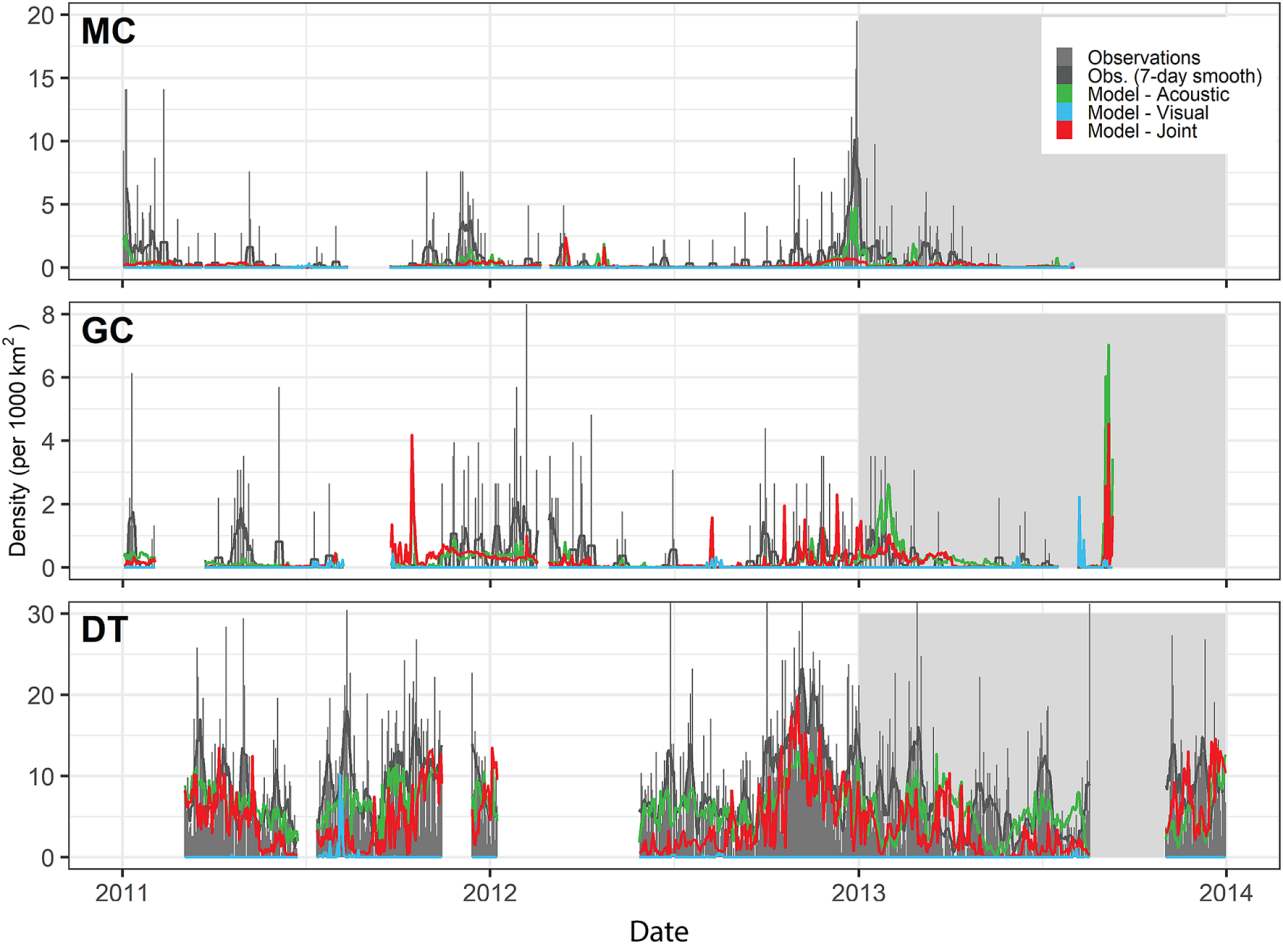
Time series of daily observed and neural network model-predicted Cuvier’s beaked whale densities from the passive acoustic monitoring sites MC, GC, and DT. Gray bars indicate observed daily density estimates. Lines indicate neural network model predictions (Blue: Acoustic only, Green: Visual only, Red: Joint) on training (white background) and test (gray background) datasets. The acoustic only model best predicted the test data based on RMSE. Time series gaps represent periods when no PAM data was being collected at the site.

Average encounter rates and density estimates for Cuvier’s beaked whales from visual surveys and PAM are summarized in Table 2. Estimated average density of Cuvier’s beaked whales from visual survey data was lower than for passive acoustic data, and this may be related to the difficulty of sighting this cryptic species. The acoustic only NN best predicted the joint test dataset based on the evaluation metric (RMSE = 3.64; Table 3). The best fitting GAM was the joint model (RMSE = 3.77). The most important variables in the NN models were SST, SSH, chlorophyll A, salinity and distance to anticyclonic eddies (Fig. 3, Supplementary Table 5). Current magnitude and mixed layer depth were influential in the GAM, but less so in the NN. The influence of chlorophyll A in the GAM was very limited, and eddies were excluded as having no significant effect. The acoustic-only model best predicted the acoustic time series according to the RMSE metric (Fig. 4) in the test dataset. Introduction of the visual data introduced spikes in time series predictions, likely due to low sighting rates which may have contributed to overfitting of the few non-zero observations in both the GAM and NN predictions. However the inclusion of the visual data in the joint NN predicted lower density estimates in the central GOM where no sightings occurred, as compared to the acoustic-only models and joint GAM (Fig. 4, Supplementary Fig. 8).

**Figure 5:**
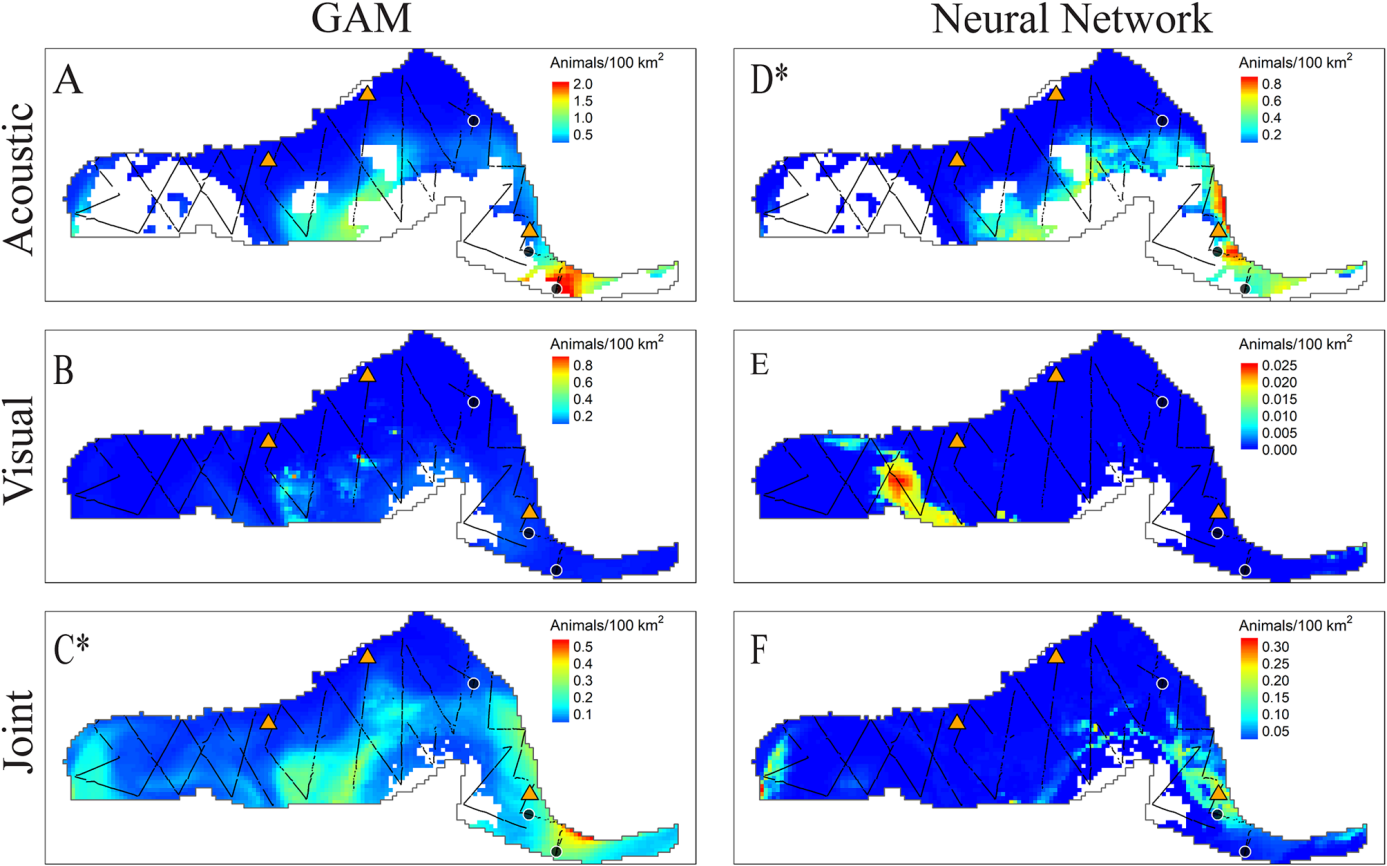
Predicted density (colormap) of Cuvier’s beaked whale across the model area for July 2009 using GAMs (subplots **A**–**C**) and NNs (**D**–**F**). Models were trained on acoustic data only (subplots **A** and **D**), visual data only (subplots **B** and **E**), and the joint dataset (subplots **C** and **F**). The 2009 NOAA cruise was withheld from training for model testing purposes and is shown with track lines overlaid (black lines). Black dots indicate the locations of Cuvier’s beaked whale sightings during this cruise. HARP locations are indicated as orange triangles. Whitespace within the model region (delimited by a gray line) indicates that no predictions were made because values of one or more predictor variables in the test data fell outside the range of observed values in the training set. An asterisk indicates the best fitting model based on RMSE (subplot **D**, the acoustic-only NN in this case). Maps created using tmap^45^.

Most models predicted highest densities in warm, salty waters near mesoscale eddy features (Fig. 5). Beaked whales are most commonly sighted along the continental slope, however no bottom depth or topography metric was included in the models. Nonetheless, neural network models had higher predictions for areas where Loop Current features interacted with the continental slope (Fig. 5), and this coincided with the locations of beaked whale observations in the visual test set. The acoustic-only model may over-estimate predicted densities because all monitoring sites used for model training were located on the continental slope, and the DT site had unusually high encounter rates. In the 2009 test set, observed salinity in the western GOM, and SSH values within the Loop Current and some eddies fell outside of the observed ranges in the acoustic-only training set. These regions are represented as gaps in the acoustic prediction maps. In the joint maps, these gaps are filled because they fell within observation ranges of the visual training data.

#### Sperm whale

**Figure 6:**
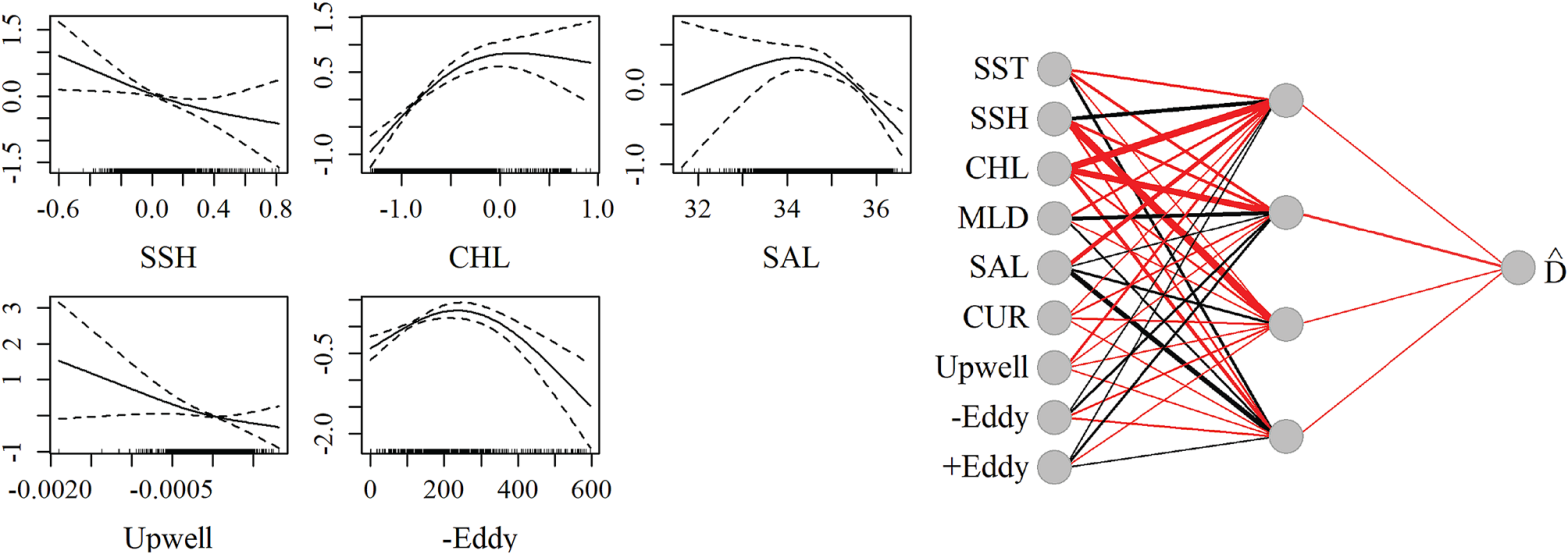
Influence of predictor variables in the best fitting sperm whale density models. *Left*: Smooths of significant terms in the joint GAM. *Right*: Neural network for sperm whale joint density model. Annotations as in Fig. 3.

Average encounter rates and density estimates for sperm whales from visual surveys and PAM are summarized in Table 2. The joint NN best predicted the joint test dataset based on the evaluation metric (RMSE = 5.41; Table 3), and the best fitting GAM also was also the joint model (RMSE = 5.61). The most influential predictor variables in the network were SST, SSH, chlorophyll A, salinity and distance to both cyclonic and anti-cyclonic eddies (Fig. 6, Supplementary Table 5). The GAM included cyclonic eddy distance as a significant factor, and did not include SST or anticyclonic eddy distance.

**Figure 7:**
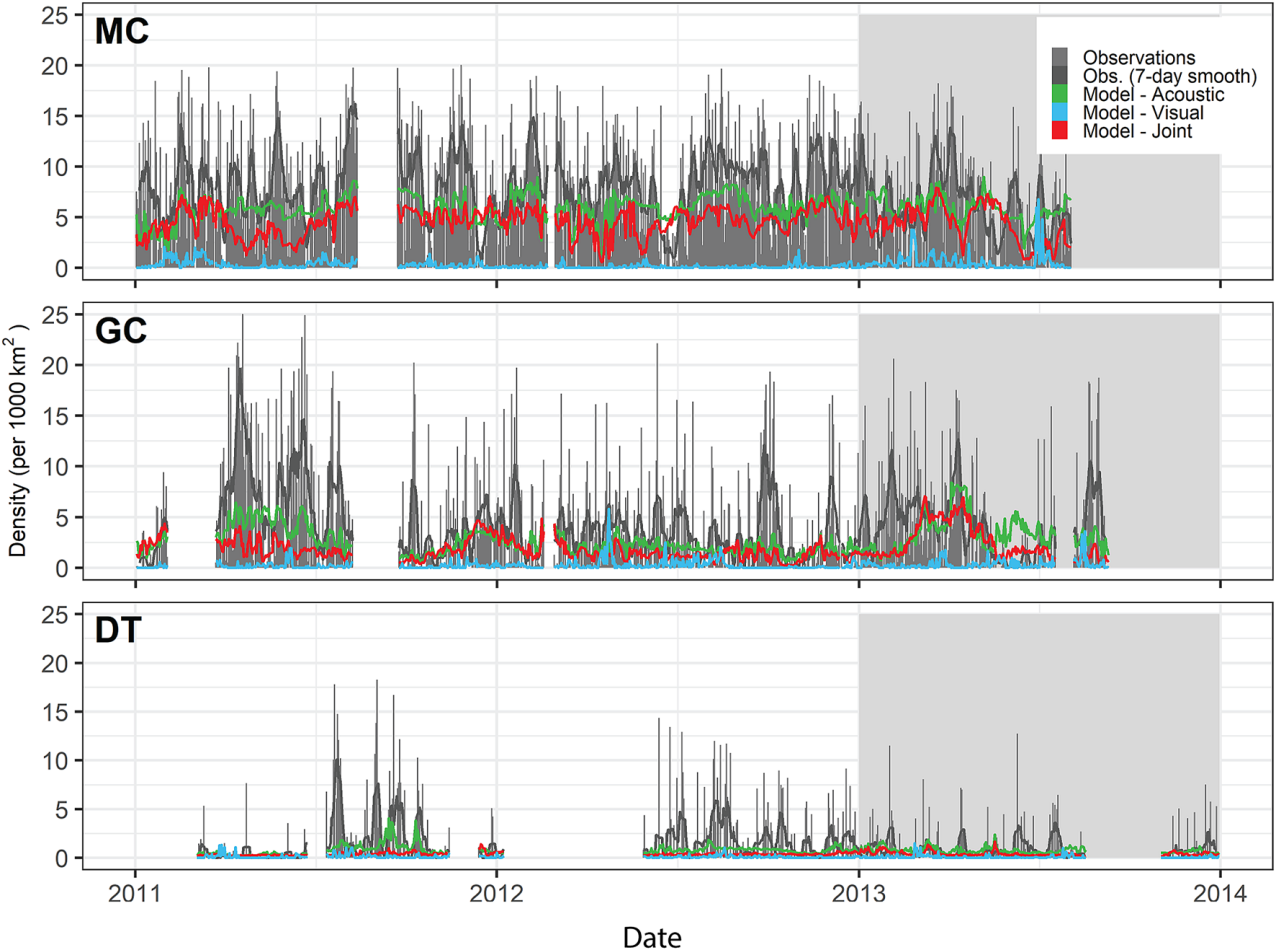
Time series of daily observed and neural network model-predicted sperm whale densities from the passive acoustic monitoring sites MC, GC, and DT. Markings as in Fig. 4.

**Figure 8:**
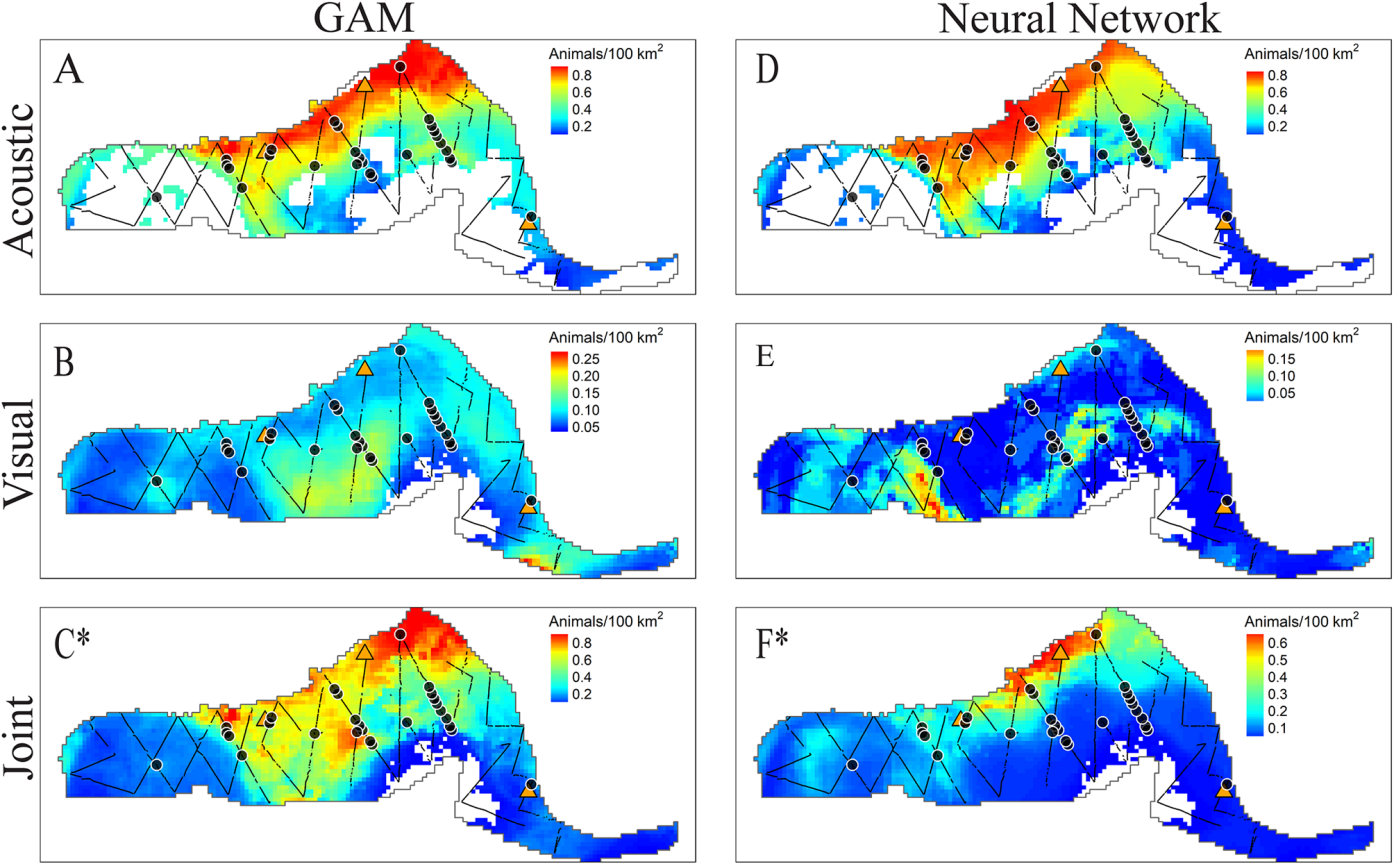
Predicted sperm whale densities (colormap) across the model area for July 2009 from density-based GAMs (subplots A-C) and NNs (D-F). Annotations as in Fig. 5. The best fitting model based on RMSE was the joint neural network. Maps created using tmap^45^.

Sperm whales were detected at high rates at the two northernmost PAM sites (Fig. 7), and models based on acoustic data for sperm whales predicted high densities throughout the north-central GOM where oceanographic conditions are affected by Mississippi River outflow. However this pattern was not reflected in model predictions from visual data alone, which predicted higher densities in deep waters adjacent to the Loop Current (Fig. 8, Supplementary Fig. 9). In the 2009 visual survey data, a concentrated string of sperm whale sightings occurred along one track line. Elevated densities and encounter probabilities were predicted in that region by the acoustic models and the joint GAM, but no model predicted such a high concentration relative to other areas. Of all models the joint GAM seems to provide the most credible spatial match with the 2009 sightings data, although it is not the best fitting based on RMSE overall. The lack of strong association between GOM sperm whale densities and loop current eddies suggested by the acoustic-only and joint models is consistent with prior analyses^6^.

#### Risso’s dolphin

**Figure 9:**
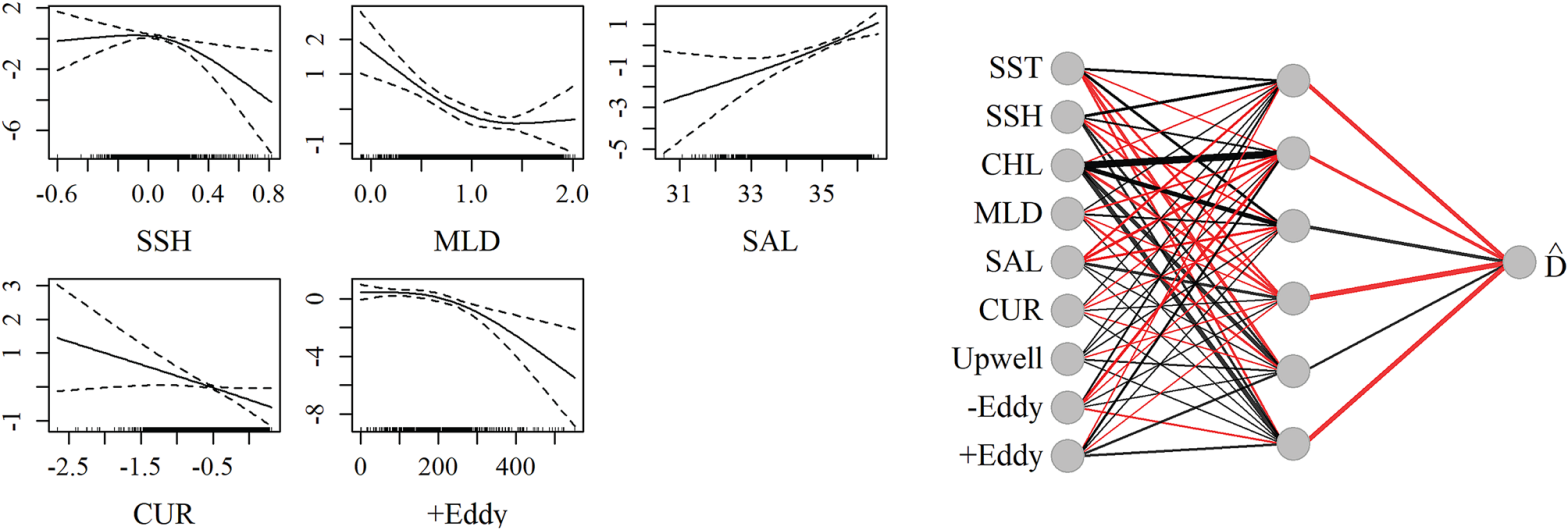
Influence of predictor variables in the best-fitting Risso’s dolphin density models. *Left*: Smooths of significant terms in the joint GAM. *Right*: Relative influence of input nodes in the acoustic-only NN. See Fig. 3 for details.

**Figure 10:**
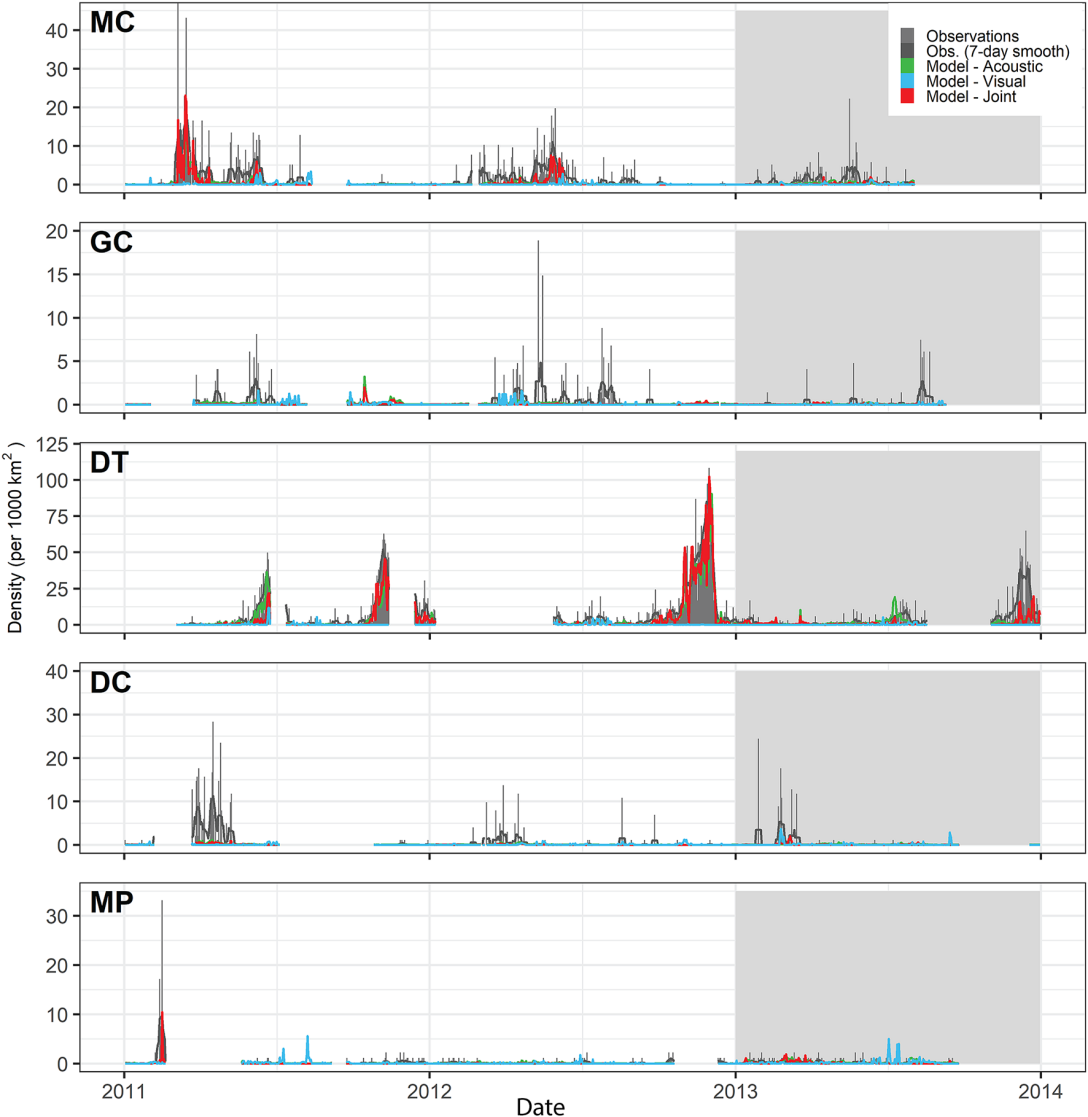
Time series of daily observed and neural network model-predicted Risso’s dolphin densities from the passive acoustic monitoring sites. Markings as in Fig. 4.

**Figure 11:**
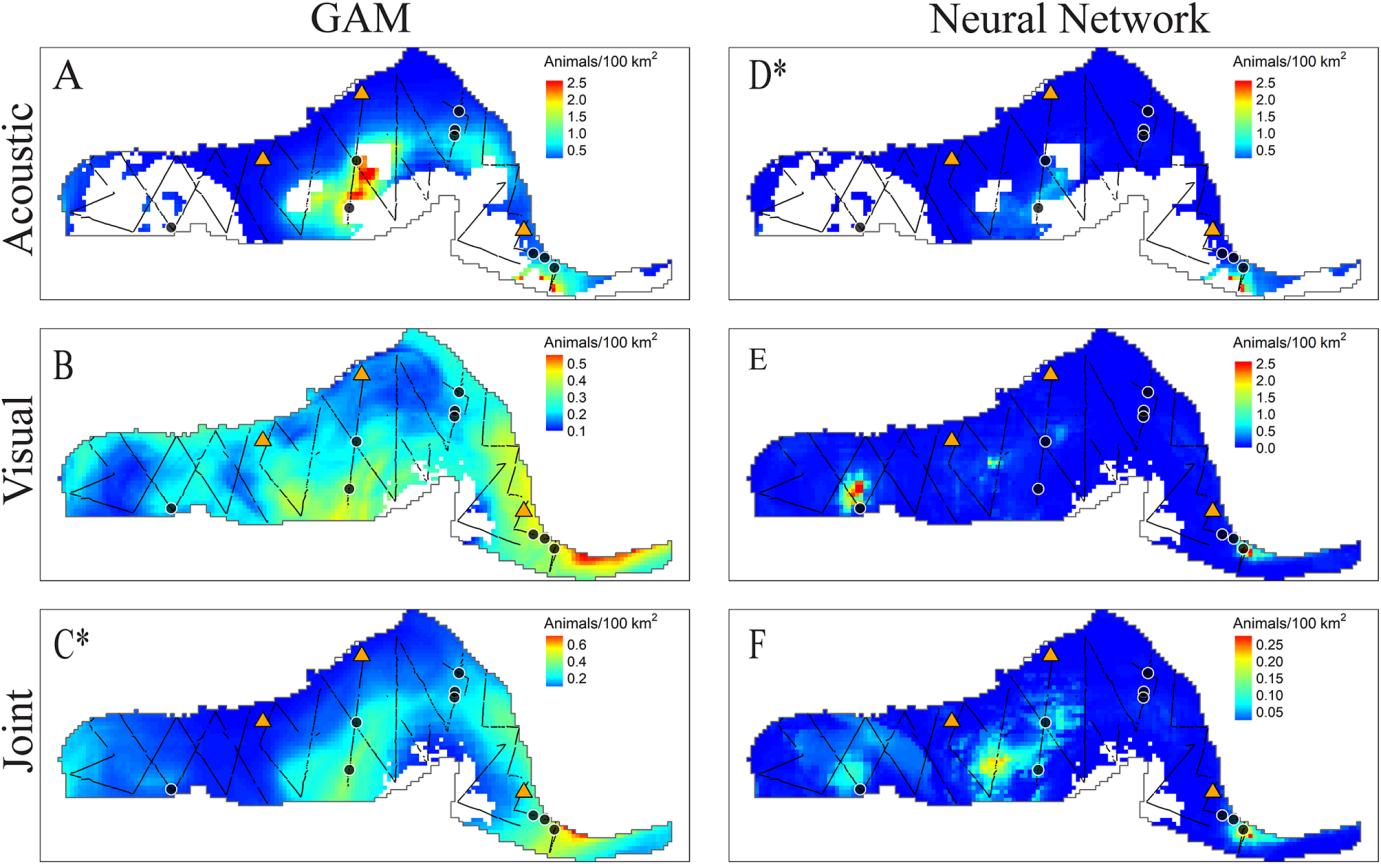
Predicted Risso’s dolphin densities (colormap) across the model area for July 2009 from GAMs (**A**–**C**), and NNs (subplots **D**–**F**). Subplots from top to bottom represent acoustic-only (**A**, **D**), visual-only (**B**, **E**), and joint (**C**, **F**) model predictions. Note that colorbars vary between visual-only and other models, due to somewhat large differences in the magnitudes of predictions. Annotations as in Fig. 5. Maps created using tmap^45^.

Average encounter rates and density estimates for Risso’s dolphins from visual surveys and PAM are summarized in Table 2. Although Risso’s dolphins are considered deep divers, they frequent shallower waters than the other species, therefore acoustic density estimates from the two shallow PAM sites (DC and MP) were included in the acoustic only and joint models. Risso’s dolphins were irregularly sighted and detected in high densities, with overall low occurrence otherwise, creating a challenge for model fitting. The best fitting models based on RMSE were the acoustic only NN and the joint GAM (RMSE = 17.72 and 17.63 respectively; Table 3). Important predictors in both models included SSH, salinity, and distance to cyclonic eddies (Fig. 9, Supplementary Table 5). The GAM additionally included mixed layer depth and salinity, while the NN included SST, chlorophyll A and distance to anti-cyclonic eddies. The predicted distributions are consistent with prior work suggesting distance to eddies and SST as important predictors of Risso’s dolphin occurrence^46,47^. A seasonal signal is apparent in the acoustic data, with summer occurrence at three of the northernmost monitoring sites (MC, GC, and DC), and mixed, but predominantly winter, occurrence at the southernmost site (DT; Fig. 10). This signal is not reflected in the visual model predictions which relied on summer surveys. Acoustic density estimates at DT were three to four times higher than at other sites, leading to some model overfitting in the case of the NN, which predicted small regions of high density in both the acoustic only and visual only models (Fig. 11, Supplementary Fig. 9) around mobile eddy features. In this case, the overfitting protections of the GOM implementation helped produce more generalized spatial predictions. The joint and visual only models all correctly predicted a hotspot in the western GOM, however the visual-only GAM broadly predicted a more widespread spatial distribution than observed. All models suggested a strong association with Loop Current eddy features.

## Discussion

Cetacean visual survey and PAM methods have complementary strengths. By transiting across the entire study area, the visual surveys provide valuable spatial information including observations across mesoscale features such as eddies, fronts and currents. PAM provides observations for the full range of seasonal conditions, including changes in temperatures and productivity. By combining the two datasets, expanded ranges of the predictor variables were available. However, certain variables which might have been useful for improving predictions, including bathymetry, season and anthropogenic noise, were ultimately excluded because one or the other method did not adequately sample them. Adding these additional drivers as well as larger, more variable training sets will be necessary to better identify functional relationships between habitat descriptors and GOM marine mammals. Models could likely be improved in the future by incorporating additional PAM sensors for better spatial coverage, adding additional spatial parameters, and by including additional visual survey data collected during fall and winter months. It is important to note that the conditions used to generate the summer predictive map were averages from July 2009, however the survey spanned from June 17 to August 9 2009, proceeding from east to west, and back to east. Oceanographic features such as eddies and fronts in the eastern GOM may have shifted between the time of the visual effort and the time represented in Figs. 5, 8, and 11.

Models trained on acoustic data alone were effective for predicting offshore distributions; however, they tended to overemphasize the influence of conditions observed at specific monitoring sites and predicted higher encounter rates and densities in offshore regions than was expected based on the visual survey data. Offshore oceanographic conditions were not completely represented in the range of conditions observed at the PAM sites, particularly for the western GOM. Visual-only models tended to predict lower densities than expected at the acoustic monitoring locations based on the acoustic data, and were prone to overfitting due to low sighting rates, as in the Cuvier’s beaked whale case. In cases where observation sample sizes are small, evaluating the effectiveness of the models becomes difficult. One option for improved acoustic model evaluation in the future may involve aggregating sightings data for cryptic species over multiple years.

RMSE is an imperfect metric of goodness of fit in this context, but it is reported to provide a quantitative comparison. RMSE goodness of fit estimates are also impacted by the ratio of test observations of each datatype, and the magnitudes of the residuals from the models. It may be more informative to compare the predictions of the models qualitatively, noting where they agree or disagree with each other and with the test data, as well as regions of high and low confidence. It appears likely from this pilot study that different model types will predict better depending on the species, coverage of the data available, and confidence associated with the observations. Future modeling efforts may benefit from considering the predictions of a variety of models as an ensemble, rather than relying on one modelling strategy as the “best”.

Joint models present a number of challenges which are illustrated by this work but not fully solved here. For instance, proponents of either survey modality will be concerned about the relative importance of either data type on joint model predictions. Additionally, each observation carries its own uncertainty, and this should likely be incorporated in future modeling efforts, however reconciling uncertainties across modalities is challenging and likely controversial. In this study we used similar numbers of observations of either type in the training sets, and assumed equal confidence across all observations, but future work will need consider more advanced strategies. Optimally, appropriate weights could be determined empirically for each species using simultaneous, co-located visual and acoustic surveys^48^. A likelihood of visually observing or acoustically detecting a group of animals, given that the animals were present, could potentially derived, but it would need to consider numerous factors including time of day, sea surface conditions, and ambient noise levels.

Alternatives to models trained simultaneously on both data types may ultimately prove more interpretable. One approach, illustrated here and in other studies^49^, is to use one survey modality to validate predictions of models trained with another. Another option is to average the density predictions of independent models, which could be considered a simple form of ensembling. We considered the latter approach, however averaging introduced undesirable artifacts and discontinuities in cases where one or the other model did not have coverage. Future research may resolve this by incorporating more acoustic monitoring locations and year round visual survey data.

NOAA shipboard surveys also collect passive acoustic recordings using towed hydrophone arrays that could be used to detect certain species missed by observers, including sperm whales which perform long dives (mean duration = 45.5 min^50^) that can exceed the duration of a 10 km transect transit (≤ 32.4 min), during which they are not available for sighting by visual observers (and, therefore, not accounted for by double-blind g(0) estimation methods) but are readily detected acoustically. Aerial visual surveys could also be incorporated where available.

The number and variety of acoustic monitoring locations is clearly a serious limitation of the present study and efforts to expand the dataset are underway. Nonetheless, the results of this limited pilot study suggest that PAM data is effective for predicting cetacean distributions across broad oceanographic regions, in part because of the mobility of oceanographic features. Additional insights may be achieved by combining PAM and visual survey data to leverage the temporal and spatial coverage strengths of the respective methods, which can fill in gaps in spatiotemporal predictions for large offshore regions. Joint model predictions did not merely reflect the average of the visual survey and PAM model predictions methods. Instead, in some cases the predicted distributions were refined by running the learning algorithm on the combined datasets. Visual survey data was useful for validating spatially extrapolated acoustic model predictions, and acoustic time series indicated weaknesses in visual survey-based models when those models were used for temporal predictions, or under during conditions unlike those observed during spring and summer surveys available for this study. Due to the limitations of both datasets, some species’ habitat preferences may have been incompletely sampled or missed entirely.

Group-based density estimation in passive acoustics only considers the probability that at least one animal in a group is acoustically detected, and uses an average group size to estimate density, rather than relying on estimates of individual echolocation click detectability to count individual animals. Sperm whales and Cuvier’s beaked whales are generally observed in small groups, however, high variability in the Risso’s dolphin PAM time series may reflect high variability in group sizes (median: 8 animals/group, high: 96 individuals/group in this visual survey dataset). In such cases, the use of an average group size may not adequately capture true patterns in species density, and cue based acoustic methods may be more appropriate in future delphinid distribution modeling efforts. Optimally, group sizes could be determined from the acoustic data directly, rather than relying on visual group sizes as a proxy for acoustic group size. Efforts to develop methods for this are ongoing, but they are likely to be most uncertain for species found in large groups.

GAMs are widely-used for cetacean habitat modeling, and they performed well in this case study using relatively simple implementations. NNs proved to be a promising alternative solution with greater fitting flexibility and ability to learn unspecified interactions. The consideration of interactions between environmental predictors using NNs appeared to improve predictions in many cases. However, unlike GAMs, NNs do not rely on distributions to fit the data, and this appeared to make them susceptible to overfitting, particularly in cases where the response variable contained large numbers of zeros or extreme values. Introducing uncertainty estimates associated with the observations could alleviate some of overfitting issues. Although neural networks are often thought of as “black boxes”, the single hidden layer networks trained in this study facilitated the use of relatively straightforward methods for interpreting variable importance and interactions. Relative importance assigned to the various environmental drivers was generally comparable between the GAMs and NNs. Fit flexibility could be increased in the GAMs by increasing the dimension of the basis of the splines for some predictors. However, in this study, higher dimensions resulted in poorer predictions due to overfitting at the data-poor edges of covariate distributions. Interaction terms could be incorporated into the GAMs if explicitly specified by the user based on prior knowledge of the system, or added based on insights from the NNs. GAMs were much faster to train than the NNs, due to the NN averaging approach which required many training iterations, and due to the cost of testing different hidden layer sizes for each model. NN predictions were relatively insensitive to changes in hidden layer size over the range of sizes tested, although high and low predictions tended to become more defined as hidden layer size increased. Mean-squared error metrics heavily penalize large discrepancies between predictions and observations; therefore, the evaluation metric used to select the optimal hidden layer size may have favored somewhat under-fitted NN models which predicted mid-range values. Both the visual survey and PAM datasets are fairly stochastic, such that two observations with seemingly identical environmental conditions can have very different response values, leading to penalties from the evaluation metric for learned associations.

This case study represents a step forward in efforts to produce high-confidence, year-round, wide-scale predictive models for cetacean species distributions by combining visual survey and PAM data. The methods can be extended to include additional data from shipboard visual surveys and PAM, as well as data from other platforms such aerial surveys (manned or autonomous), and hydrophone-equipped autonomous underwater vehicles, to incorporate a more thorough set of observations across open ocean regions. These data were collected following the Deepwater Horizon Oil spill. Long-term declines in density of some odontocete species in the GOM may have occurred^16^. The duration of the time series used for this study was limited to a 3-year period during which density estimates appeared to be generally stationary at the PAM sites. Visual survey data were collected over a longer period, but no clear inter-annual trends were observed in the data. As longer time series are incorporated, additional methods may be needed to handle long term changes in stock sizes. Joint modeling methods and methods that consider ensembles of models trained on a variety of data types may be useful for improving the understanding of visually cryptic and rare species, provided that the species’ vocalizations are acoustically distinct.

## Conclusion

Monitoring large marine ecosystems is resource intensive, and few examples of monitoring programs capable of providing the spatiotemporal resolution needed by managers currently exist. PAM data can be used to improve predictions of cetacean spatial and temporal occurrence relative to predictions made by models trained on visual survey data alone. Combining observational data across multiple monitoring methods may provide a tractable solution for improving model predictions, to support estimation of offshore distributions. In this pilot study, distribution models trained using combined datasets from shipboard visual surveys and fixed PAM were able to expand and validate spatiotemporal predictions of occurrence of three odontocete species.

## Supplementary Information

Supplementary Information.
