# Supplementary material for: Cetacean distribution models based on visual and passive acoustic data
Source: Sci Rep. 2021 Apr 15;11:8240. doi: 10.1038/s41598-021-87577-1 (PMC8050100; doi:10.1038/s41598-021-87577-1)
Supplement: Supplementary file 1 — Supplementary Information. [file 41598_2021_87577_MOESM1_ESM.docx]

Cetacean Distribution Models based on Visual and Passive Acoustic Data: Supplementary Information

Kaitlin E. Frasier^1^, Lance P. Garrison^2^, Melissa S. Soldevilla^2^, Sean M. Wiggins^1^, and John A. Hildebrand^1^

^1^Scripps Institution of Oceanography, La Jolla, California, United States of America.

^2^NOAA NMFS Southeast Fisheries Science Center, Protected Resources and Biodiversity Division, Miami, Florida, United States of America.

**1. Supplementary Methods**

**1.1 Data collection**

**Supplementary Table 1.** NOAA ship-board visual survey effort used in this study. Data from 2009 (*) were reserved for model testing only, all other years were used for model training.

| Start Date | End Date | Survey Days | Cumulative On-Effort Distance (km) |
| --- | --- | --- | --- |
| 6/14/2003 | 8/17/2003 | 46 | 6,985 |
| 4/15/2004 | 6/10/2004 | 44 | 6,927 |
| 6/18/2009 * | 8/9/2009 * | 34 | 4,560 |
| 6/11/2012 | 8/5/2012 | 40 | 4,146 |
| 7/5/2014 | 7/29/2014 | 18 | 2,407 |

**Supplementary Table 2.** Passive acoustic monitoring deployment locations and effort.

| Site | Lat. N | Long. W | Depth (m) | Data Start  (mm/dd/yyyy) | Data End (mm/dd/yyyy) | Duration (days) |
| --- | --- | --- | --- | --- | --- | --- |
| MC | 28^o^ 50.78’ | 88^o^ 27.91’ | 980 | 12/20/2010 | 03/21/2011 | 91 |
|  | 28^o^ 50.78’ | 88^o^ 27.95’ | 980 | 03/22/2011 | 08/15/2011 | 146 |
|  | 28^o^ 50.80’ | 88^o^ 27.99’ | 980 | 09/22/2011 | 02/21/2012 | 152 |
|  | 28^o^ 50.85’ | 88^o^ 28.04’ | 980 | 02/28/2012 | 12/11/2012 | 288 |
|  | 28^o^ 50.78’ | 88^o^ 28.06’ | 900 | 12/11/2012 | 08/03/2013 | 235 |
| GC | 27^o^ 33.47’ | 91^o^ 10.01’ | 1,160 | 11/08/2010 | 02/02/2011 | 86 |
|  | 27^o^ 33.42’ | 91^o^ 10.07’ | 1,100 | 03/23/2011 | 08/08/2011 | 138 |
|  | 27^o^ 33.43’ | 91^o^ 10.06’ | 1,100 | 09/23/2011 | 02/17/2012 | 118 |
|  | 27^o^ 33.44’ | 91^o^ 10.56’ | 1,100 | 02/28/2012 | 12/12/2012 | 289 |
|  | 27^o^ 33.35’ | 91^o^ 10.09’ | 1,100 | 12/13/2012 | 09/10/2013 | 271 |
| DT | 25^o^ 31.91’ | 84^o^ 38.25’ | 1,320 | 03/03/2011 | 07/12/2011 | 129 |
|  | 25^o^ 31.86’ | 84^o^ 38.26’ | 1,300 | 07/13/2011 | 11/14/2011 | 124 |
|  | 25^o^ 31.87’ | 84^o^ 38.27’ | 1,300 | 12/14/2011 | 01/09/2012 | 26 |
|  | 25^o^ 31.94’ | 84^o^ 38.04’ | 1,200 | 05/27/2012 | 12/7/2012 | 195 |
|  | 25^o^ 31.94’ | 84^o^ 38.05’ | 1,200 | 12/07/2012 | 8/18/2013 | 253 |
|  | 25^o^ 32.22’ | 84^o^ 38.15’ | 1,158 | 11/01/2013 | 12/31/2013 | 60 |
| DC | 29^o^ 03.13’ | 86^o^ 05.77’ | 268 | 10/21/2010 | 02/06/2011 | 108 |
|  | 29^o^ 03.21’ | 86^o^ 05.80’ | 260 | 03/21/2011 | 07/06/2011 | 107 |
|  | 29^o^ 02.89’ | 86^o^ 05.90’ | 260 | 10/26/2011 | 03/02/2012 | 144 |
|  | 29^o^ 02.89’ | 86^o^ 05.90’ | 260 | 10/26/2011 | 03/02/2012 | 144 |
|  | 29^o^ 02.88’ | 86^o^ 05.84’ | 260 | 03/03/2012 | 12/09/2012 | 282 |
|  | 29^o^ 02.90’ | 86^o^ 05.87’ | 260 | 12/09/2012 | 09/25/2013 | 289 |
| MP | 29^o^ 15.32’ | 88^o^ 17.81’ | 93 | 11/07/2010 | 02/19/2011 | 100 |
|  | 29^o^ 15.35’ | 88^o^ 17.70’ | 93 | 09/22/2011 | 03/01/2012 | 161 |
|  | 29^o^ 15.37’ | 88^o^ 17.60’ | 90 | 02/29/2012 | 11/24/2012 | 270 |
|  | 29^o^ 15.38’ | 88^o^ 17.51’ | 90 | 12/10/2012 | 09/25/2013 | 289 |

**1.2 Visual Surveys**


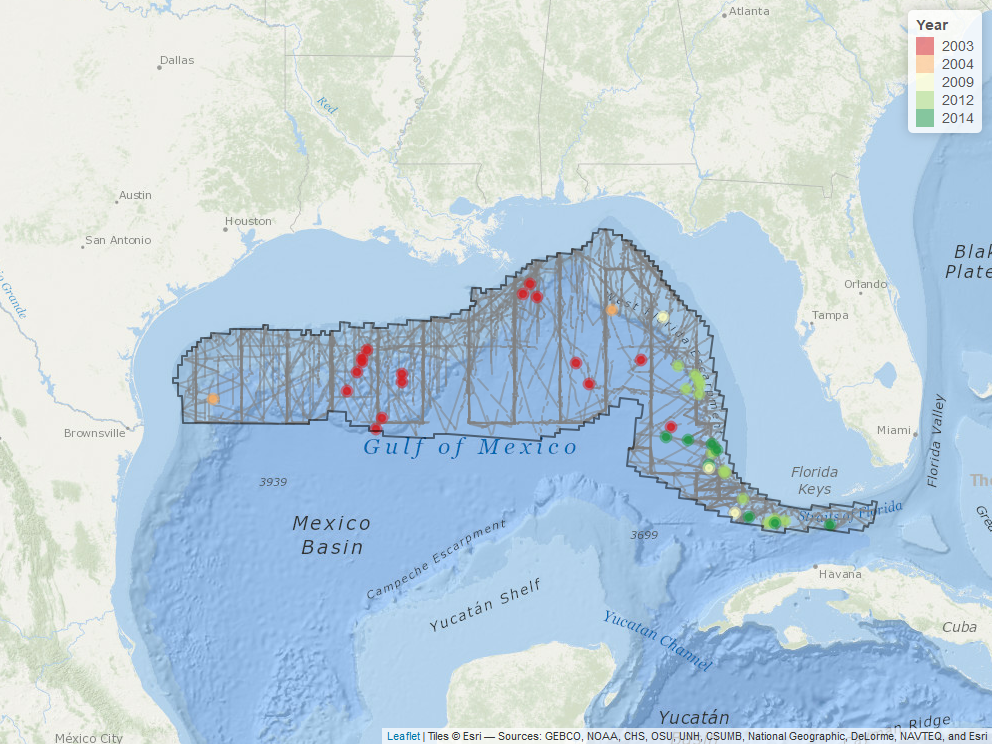


**Supplementary Figure 1.** Sightings of Cuvier’s beaked whale in the visual survey dataset. Circles on the map indicate sighting locations, with color representing year. Gray lines show survey effort, black outline delineates 200m contour and the US EEZ which bound the survey area (Map created using leafletR version 2.0.2 and mapview version 2.7.0 [1, 2]).


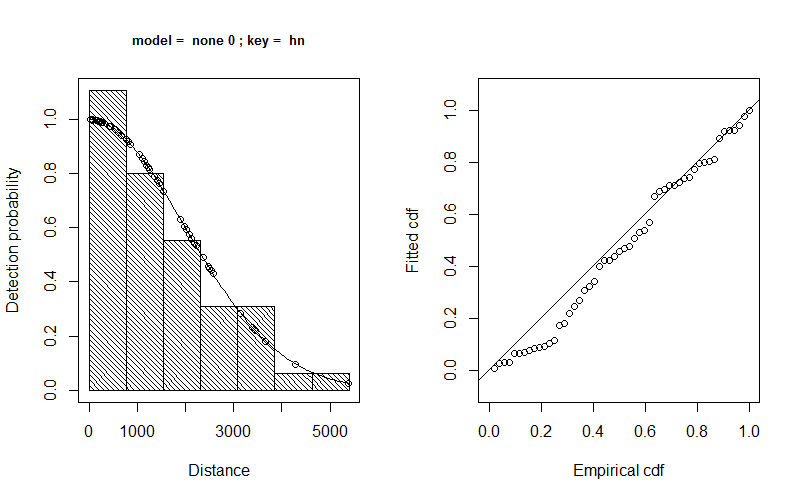
**Supplementary Figure 2.** Detection function from visual survey data for Cuvier’s beaked whales. The best fitting detection function used a half normal key.

**Supplementary Figure 3.** Sightings of sperm whales in the visual survey dataset. Markings as in Figure 1 (Map created using leafletR version 2.0.2 and mapview version 2.7.0 [1, 2]).


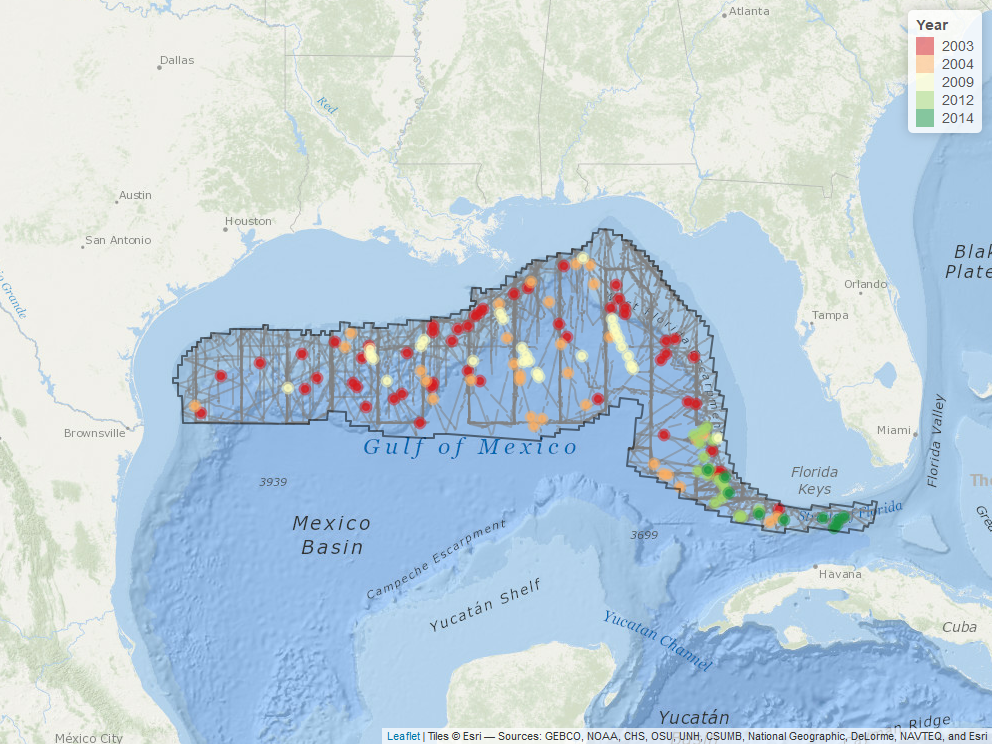


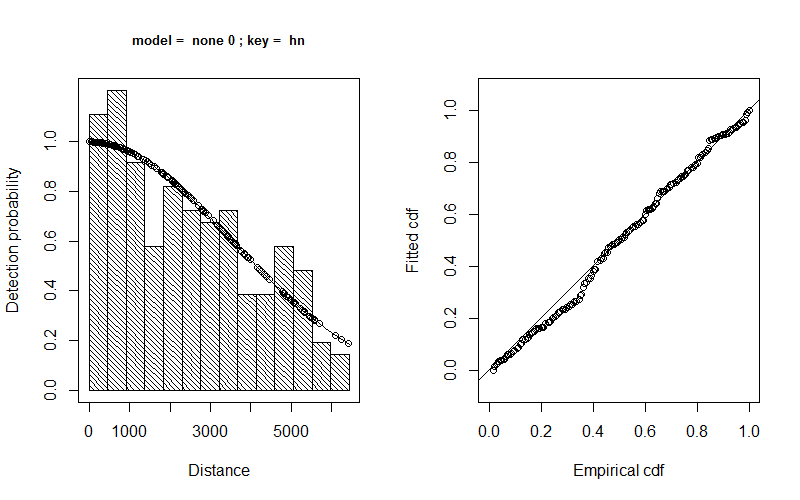


**Supplementary Figure 4**. Detection function from visual survey data for sperm whales. The best fitting detection function used a half normal key.


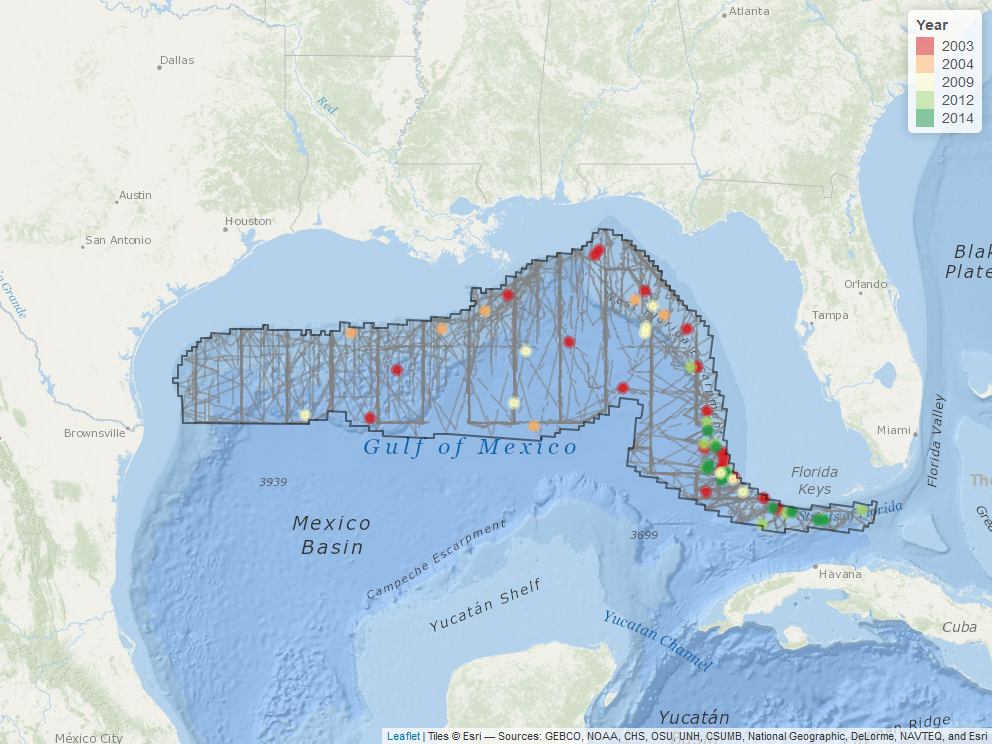


**Supplementary Figure 5.** Sightings of Risso’s dolphins in the visual survey dataset. Markings as in Figure 1 (Map created using leaflet version 2.0.2 and mapview version 2.7.0 [1, 2]).


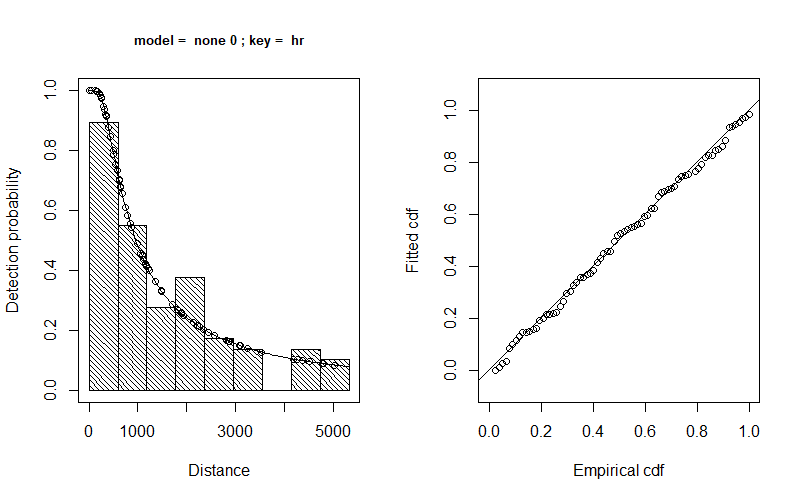


**Supplementary Figure 6.** Detection function from visual survey data for Risso’s dolphins. The best fitting detection function used a hazard rate key.

**1.2 Environmental Covariates**


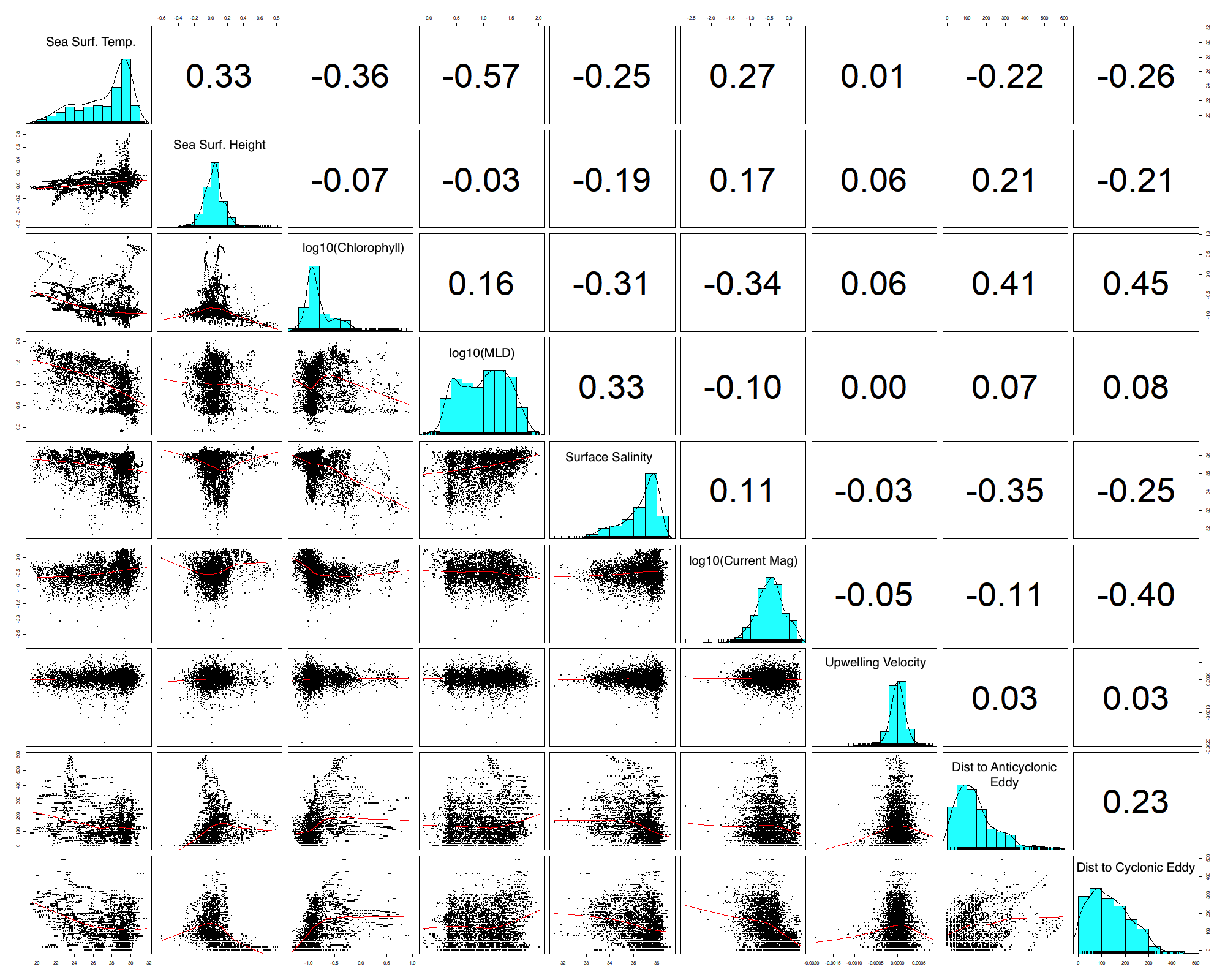


**Supplementary Figure 7.** Correlations between the nine environmental variables in the combined visual and acoustic training set. This figure is used to check for strong correlations between predictor variables which might necessitate excluding one or the other from the models. Histograms along the diagonal show the distribution of each variable. Log transformations are noted where applicable. Bivariate scatterplots below the diagonal show the relationships between paired variables, with red line indicating of paired variables, with the red line indicating a LOESS smoothed fit. Values above the diagonal represent spearman correlation coefficients calculated for each pair of variables.

**Supplementary Table** **3.** Constants used for computing visual density estimates by species using equations 1 and 2.

| **Species** | **g0** | **P(detection)**  **(**$\hat{\boldsymbol{P}}$**_vis_)** | **Truncation Distance**  **(**$\boldsymbol{w}$**; km)** |
| --- | --- | --- | --- |
| Cuvier’s beaked whale | 0.29 | 0.46 | 5.4 |
| Sperm whale | 0.52 | 0.64 | 6.4 |
| Risso’s dolphin | 0.81 | 0.29 | 5.3 |

**Supplementary Table** **4.** Constants used for computing acoustic density estimates by species using equation 3. Maximum detection range represents the longest detection range possible, not the range for a typical detection. More detail on these estimates can be found in [3, 4]

| **Species** | **P(detection)**  **(**$\hat{\boldsymbol{P}}$**_k_)** | **Maximum Detection Range**  **(**$\boldsymbol{w}$**; km)** | **Average Group Size**  **(**$\hat{\boldsymbol{s}}$**)** | **P(vocal)**  **(**$\hat{\boldsymbol{P}}$**_k_)** |
| --- | --- | --- | --- | --- |
| Cuvier’s beaked whale | 0.36 | 4.0 | 1.95 | 0.471 |
| Sperm whale | 0.50 | 12.0 | 2.65 | 0.254 |
| Risso’s dolphin | 0.40 | 5.0 | 7.0 | 1 |

**Supplementary Table 5.** Estimated relative importance of predictor variables in best-fitting GAM and neural network (NN) models. For GAMs, F-test statistics and associated p-value ranges are shown, with asterisks denoting significance codes (*** = p ≤0.001; ** = p ≤ 0.01; * = p ≤ 0.05; ^+^ =p ≤ 0.1). For NNs, the mean variable importance across all trained networks is given. These statistics are not comparable between methods (GAM vs. NN).

| **Predictor Variable** | **Cuvier’s beaked whale** | | **Sperm whale** | | **Risso’s Dolphin** | |
| --- | --- | --- | --- | --- | --- | --- |
|  | GAM | NN | GAM  (density) | NN  (density) | GAM | NN |
| SST | ^***^26.1 | 11.9 | excluded | 10.2 | excluded | 10.8 |
| SSH | ^**^4.9 | 23.2 | ^**^4.2 | 26.5 | ^*^3.6 | 20.4 |
| Log_10_(CHL) | ^+^1.9 | 20.0 | ^***^28.4 | 13.5 | excluded | 19.2 |
| Log_10_(MLD) | ^***^11.5 | 3.5 | excluded | 4.6 | ^***^12.4 | 6.4 |
| SAL | ^***^66.7 | 16.3 | ^***^10.3 | 14.8 | ^***^10.8 | 15.6 |
| Log_10_(Cur) | ^***^22.5 | 3.6 | ^*^3.0 | 5.3 | ^*^2.7 | 2.8 |
| Upwell | excluded | 3.8 | excluded | 5.2 | excluded | 3.7 |
| -Eddy | excluded | 9.8 | excluded | 10.1 | excluded | 10.2 |
| +Eddy | excluded | 7.8 | ^***^11.2 | 9.7 | ^***^8.3 | 10.8 |

**2.1 Supplementary Results**


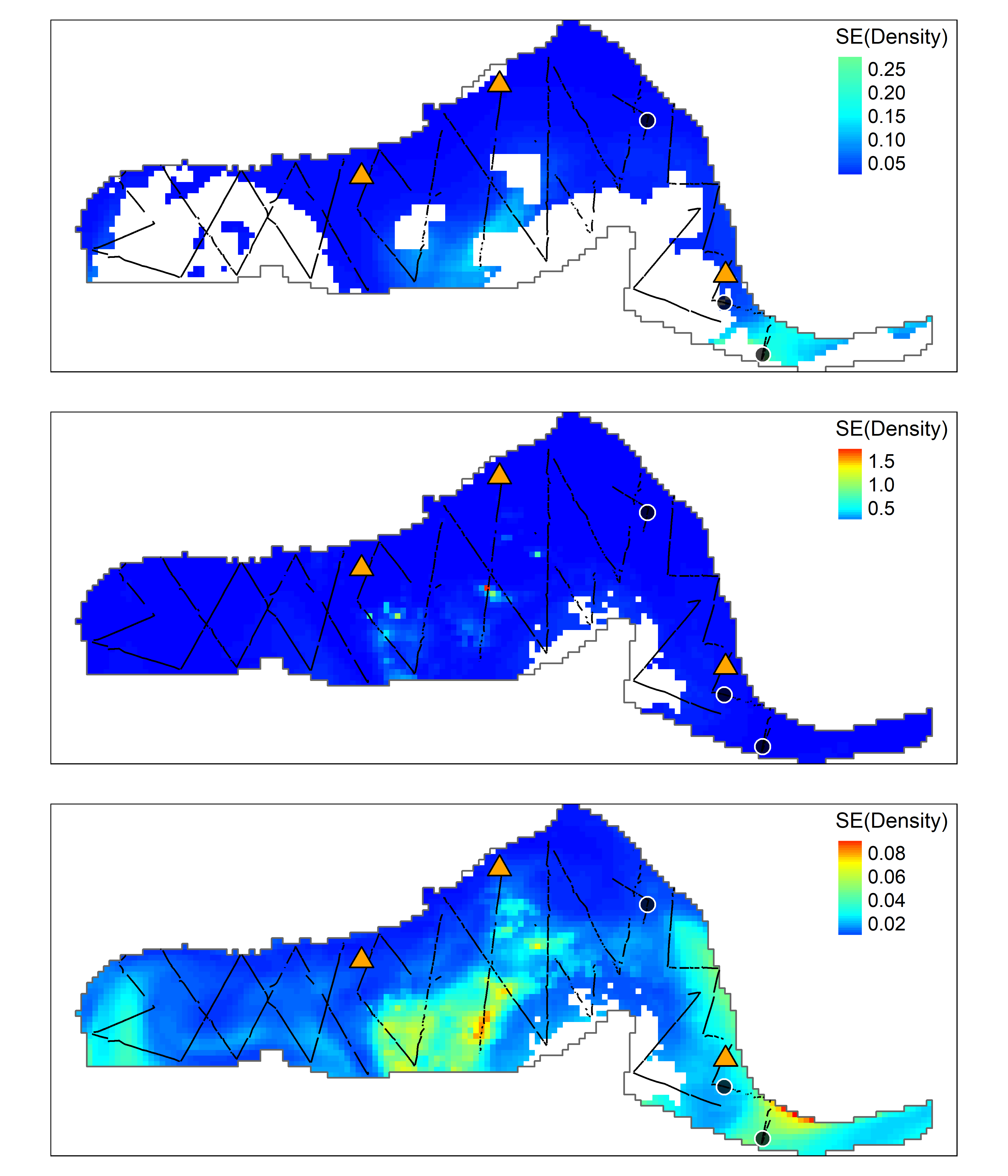

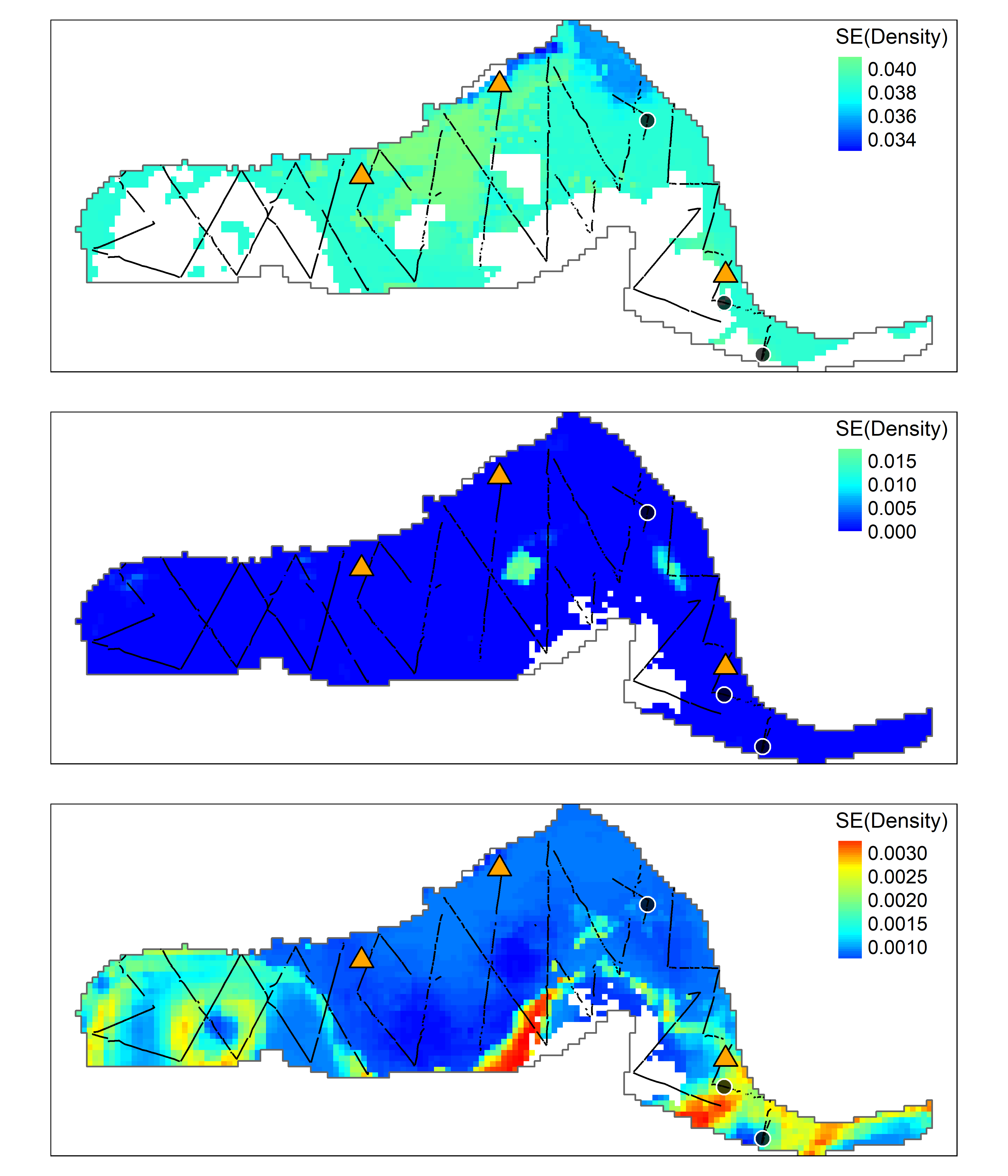
**Supplementary Figure 8.** Standard error (colormap) of predicted Cuvier’s beaked whale density across the model area for July 2009 from GAMs (A-C), and NNs (subplots D-F). Subplots from top to bottom represent acoustic-only (A, D), visual only (B, E), and joint (C, F) model predictions. Note that color bars vary between subplots. Annotations as in Figure 5. Maps created using tmap version 2.3-1 [5].

E

F

C

B

D

A

GAM

B

C

Neural Network

B

C

Acoustic

B

C

Visual

B

C

Joint

B

C

Visual

B

C

Acoustic

B

C

GAM

B

C

Neural Network

B

C


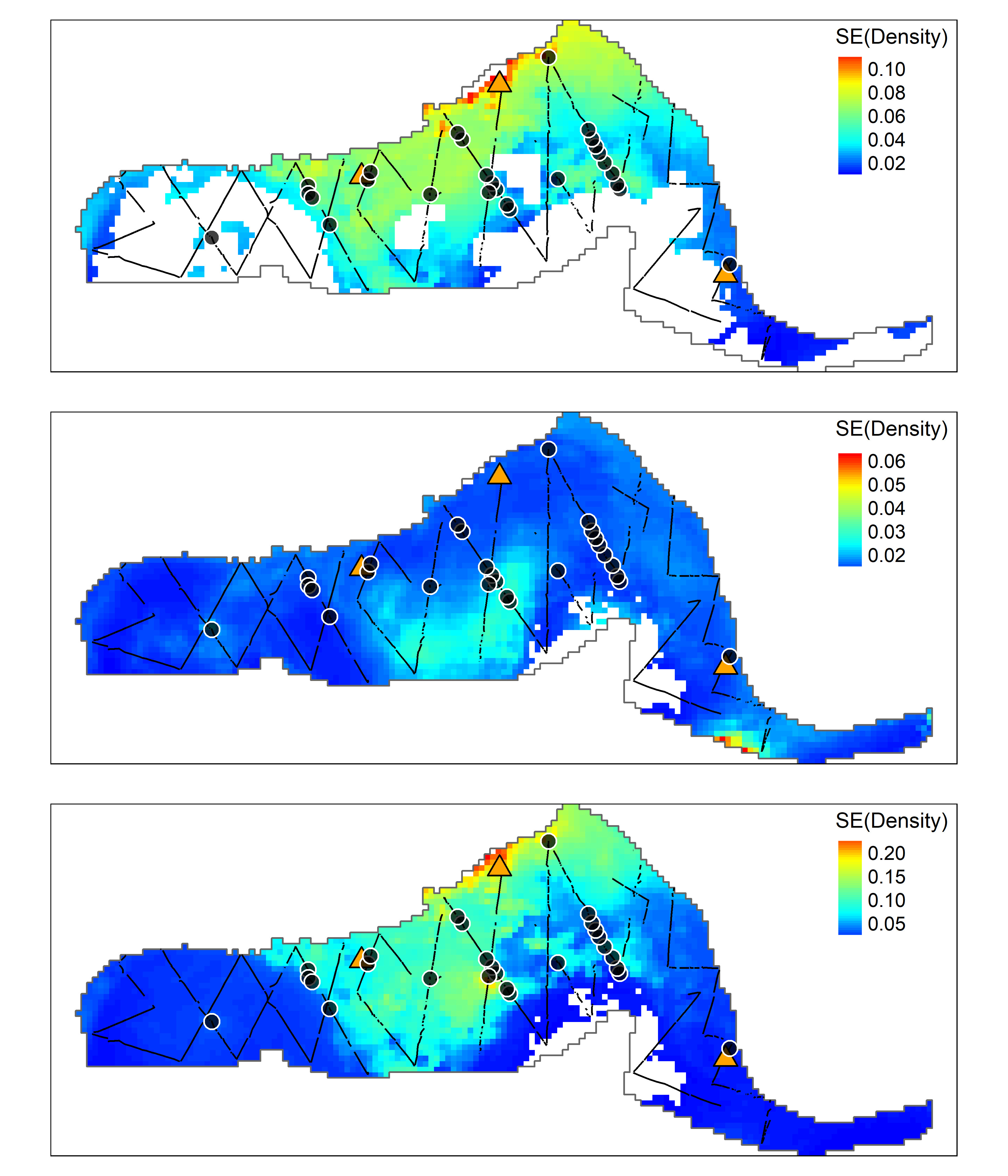

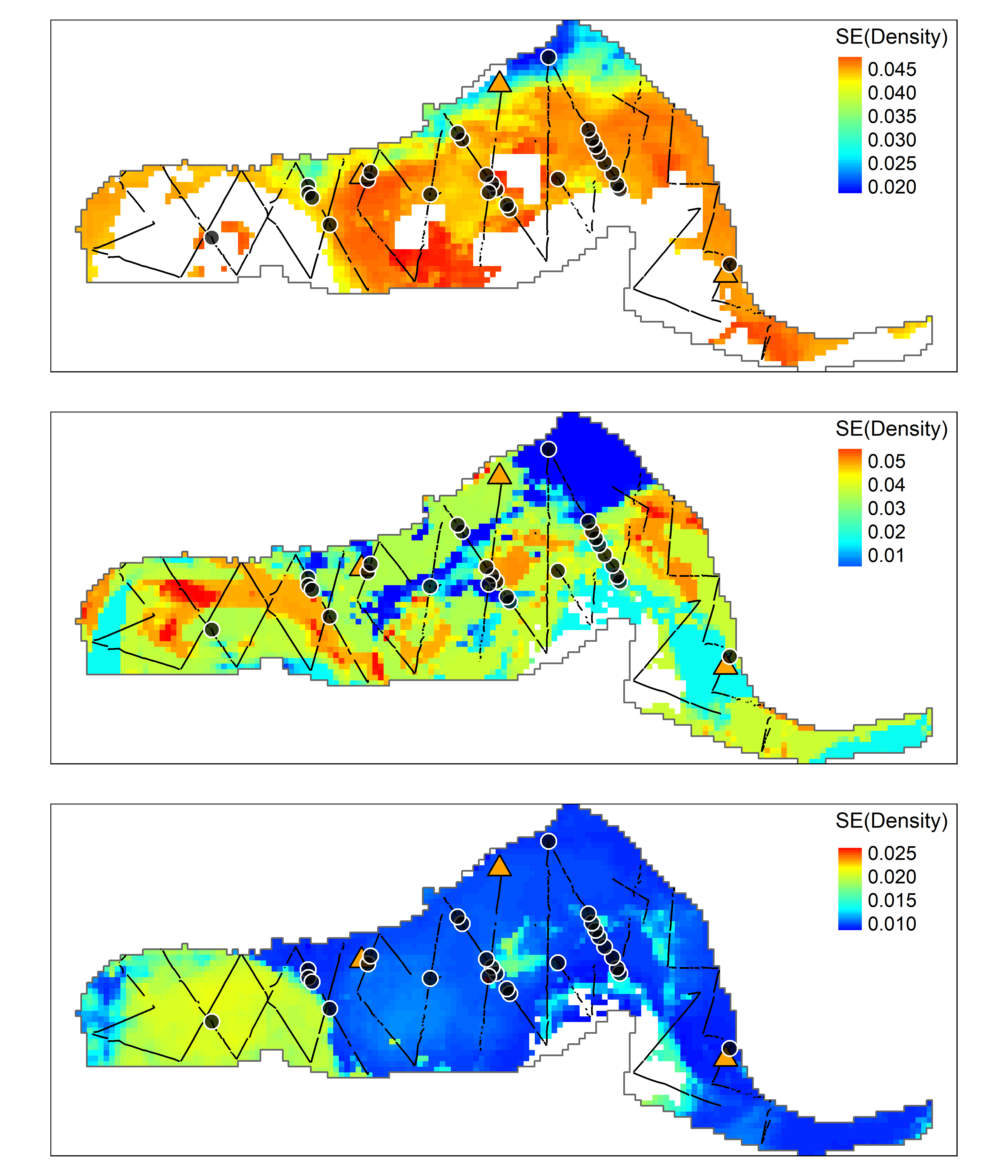
**Supplementary Figure 9.** Standard error (colormap) of predicted sperm whale density across the model area for July 2009 from GAMs (A-C), and NNs (subplots D-F). Subplots from top to bottom represent acoustic-only (A, D), visual only (B, E), and joint (C, F) model predictions. Annotations as in Figure 5. Maps created using tmap version 2.3-1 [5].

E

F

C

B

A

D

Joint

B

C


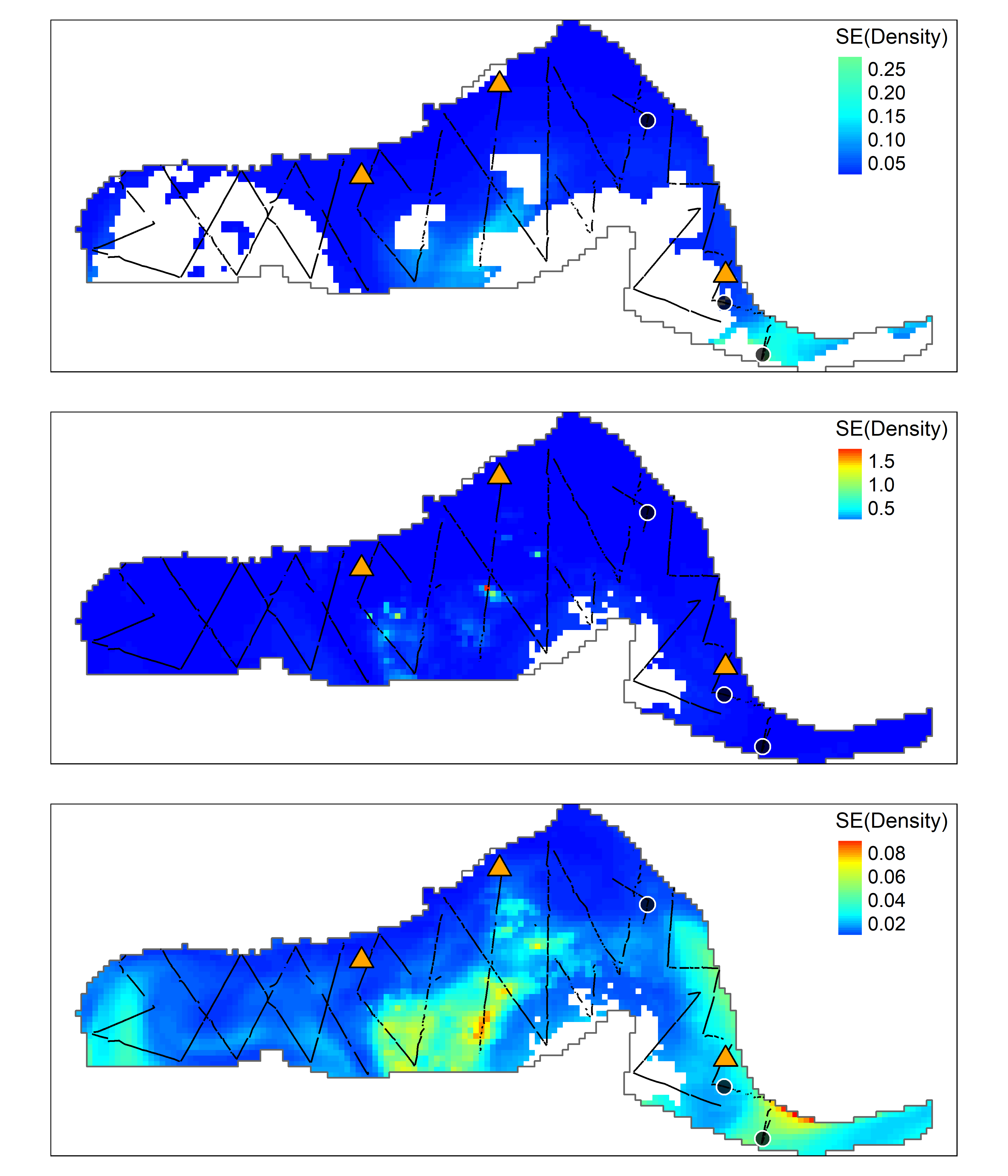

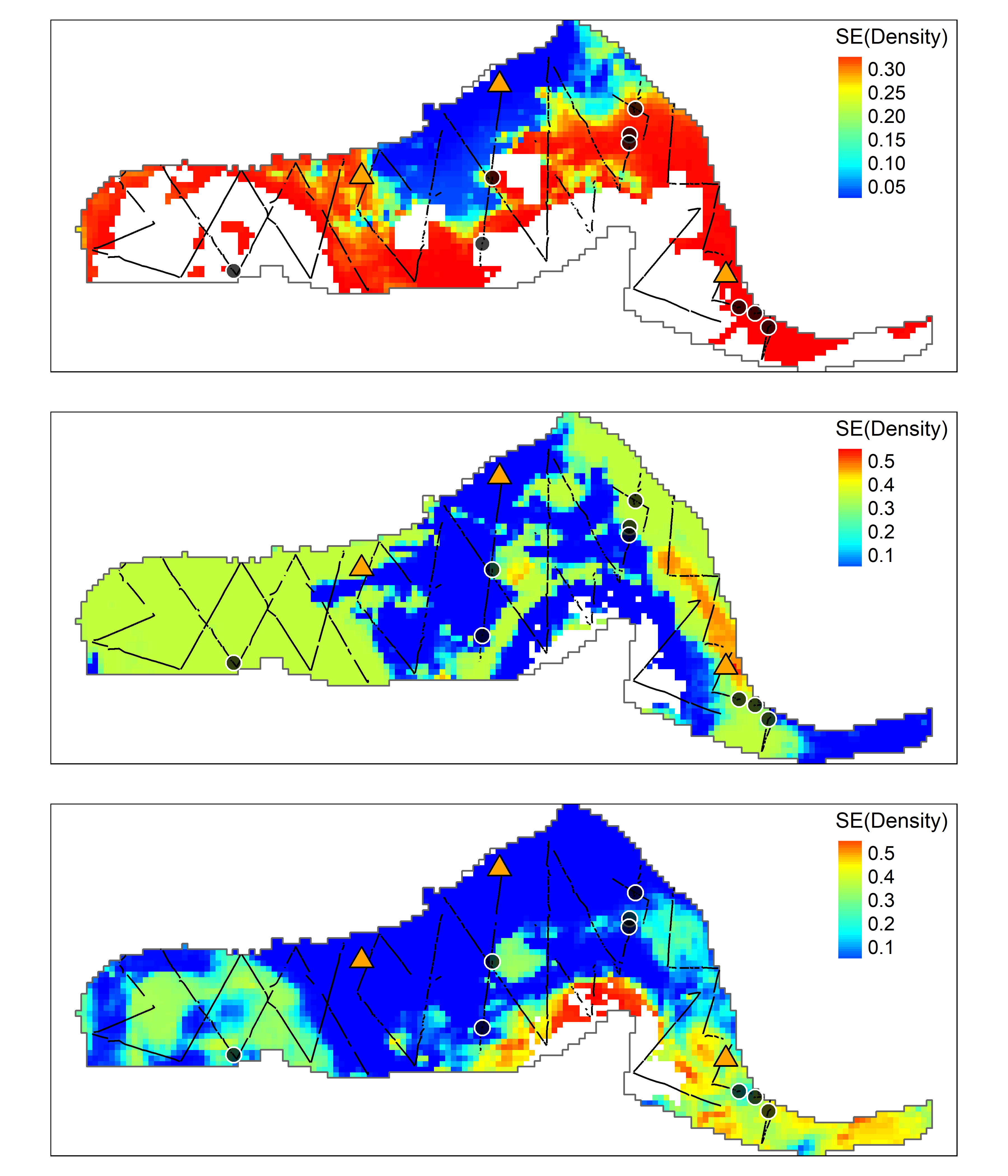


C

B

E

F

D

A

Joint

B

C

Visual

B

C

Acoustic

B

C

GAM

B

C

Neural Network

B

C

**Supplementary Figure 10.** Standard error (colormap) of predicted Risso’s dolphin density across the model area for July 2009 from GAMs (A-C), and NNs (subplots D-F). Subplots from top to bottom represent acoustic-only (A, D), visual only (B, E), and joint (C, F) model predictions. Note that color bars vary between subplots. Annotations as in Figure 5. Maps created using tmap version 2.3-1 [5].

References

1. Graul C. 2016.

2. Appelhans T, Detsch F, Reudenbach C, Woellauer S. mapview: Interactive Viewing of Spatial Data in R. R  package version 2.7.0. https://CRAN.R-project.org/package=mapview.

3. Hildebrand J, Baumann-Pickering S, Frasier K, Tricky J, Merkens K, Wiggins S, et al. Passive acoustic monitoring of beaked whale densities in the Gulf of Mexico during and after the Deepwater Horizon oil spill. Nature Scientific Reports. 2015;5:16343.

4. Frasier KE. Density estimation of delphinids using passive acoustics: A case study in the Gulf of Mexico. [Ph.D. Thesis]. La Jolla, CA: The University of California San Diego; 2015.

5. Tennekes M. tmap: Thematic Maps in R. Journal of Statistical Software. 2018;84(6):1-39. doi: 10.18637/jss.v084.i06.
